# Uncovering the Role of DNA Repair Impairment in UVA‐Induced Mutagenesis in Human Xeroderma Pigmentosum Variant Cells

**DOI:** 10.1002/mc.70028

**Published:** 2025-08-12

**Authors:** Camila Corradi, Natália Cestari Moreno, Nathalia Quintero‐Ruiz, Giovana da Silva Leandro, Marcela Teatin Latancia, Tiago Antonio de Souza, Veridiana Munford, Carlos Frederico Martins Menck

**Affiliations:** ^1^ Laboratory of DNA Repair, Institute of Biomedical Sciences University of São Paulo São Paulo SP Brazil; ^2^ Department of Cancer Biology University of Kansas Medical Center Kansas City Kansas USA; ^3^ Faculty of Applied Science Campinas University Limeira SP Brazil; ^4^ Laboratory of Genomic Integrity, National Institute of Child Health and Human Development National Institutes of Health Bethesda Maryland USA; ^5^ TauGC Bioinformatics São Paulo SP Brazil

**Keywords:** DNA polymerase eta, nucleotide excision repair, oxidative stress, pyrimidine dimers, ultraviolet A

## Abstract

Ultraviolet A (UVA) radiation induces DNA damage both directly, by forming cyclobutane pyrimidine dimers (CPDs), and indirectly, by generating oxidative stress. Cells rely on nucleotide excision repair (NER) and translesion synthesis (TLS) to tolerate these lesions. Xeroderma pigmentosum variant (XP‐V) cells, deficient in DNA polymerase eta (pol eta), exhibit a heightened risk of skin cancer due to impaired TLS. While XP‐V patients are considered NER‐proficient, our findings challenge this assumption by demonstrating that UVA‐induced oxidative stress impaired NER activity, leading to increased C > T transitions at CPD sites. Whole‐exome sequencing of UVA‐irradiated XP‐V cells revealed a substantial rise in mutations, with a distinct C > T signature characteristic of defective CPD repair. Notably, pretreatment with the antioxidant *N*‐acetylcysteine (NAC) mitigated this effect, reducing C > T transitions through enhanced NER function and decreasing C > A transversions via its antioxidant properties. These results redefine the mutagenic landscape of XP‐V cells, revealing that oxidatively generated damage to NER proteins—rather than TLS deficiency alone—contributes to their elevated mutation burden. Our findings suggest that antioxidant strategies may partially protect XP‐V patients from UVA‐driven mutagenesis enhancing the cells' DNA repair capacity, ultimately reducing skin cancer and contributing to better overall health outcomes.

**Abbreviations**
6‐4 PP: pyrimidine (6
  –4) pyrimidone
BER: base excision repair
CPD: cyclobutane pyrimidine dimer
NAC: *N*‐acetylcysteine
NER: nucleotide excision repair
NMF: non‐negative matrix factorization
PBS: phosphate‐buffered saline
ROS: reactive oxygen species
SBS: single base substitution
TC‐NER: transcription‐coupled NER
TLS: translesion synthesis
TLS: translesion synthesis
T‐strand: transcribed strand
UT‐strand: untranscribed strand
UVA: ultraviolet A
XP‐V: xeroderma pigmentosum variant

## 1 Introduction

The ultraviolet (UV) component of sunlight interacts intricately with human cells, resulting in DNA damage [1]. UV radiation spans wavelengths from 100 to 400 nm, categorized into UVC (100–280 nm), UVB (280–315 nm), and UVA (315–400 nm). While UVC is completely filtered by the ozone layer in the stratosphere, both UVB and UVA reach the Earth's surface and are recognized as genotoxic agents, classified as carcinogenic to humans [2].

Upon absorption by DNA, UVA radiation primarily induces damage to adjacent pyrimidines, leading to the formation of cyclobutane pyrimidine dimers (CPDs) [3, 4, 5], and, to a lesser extent, pyrimidine (6–4) pyrimidone photoproducts (6–4 PPs) [5]. Additionally, this portion of solar radiation indirectly harms DNA by generating oxidative stress, which enhances the oxidation of DNA bases [6, 7, 8, 9].

The harmful effects of solar radiation are prominently illustrated in patients with xeroderma pigmentosum (XP), a rare autosomal recessive syndrome characterized by extreme sensitivity to UV radiation. This condition leads to hypo‐ or hyperpigmentation and a markedly increased risk of developing cutaneous and ocular neoplasms, primarily due to defects in the nucleotide excision repair (NER) mechanism [10, 11]. XP is classified into seven complementation groups (XP‐A to XP‐G), each corresponding to a gene involved in the NER pathway [12, 13]. In the variant form of XP (XP‐V), cells display proficient NER but fail to efficiently replicate UV‐damaged DNA [14, 15, 16]. XP‐V cells are deficient in the *POLH* gene, which encodes DNA polymerase eta (pol eta), a key polymerase in translesion synthesis (TLS) [17, 18]. Pol eta enables error‐free replication of cyclobutane pyrimidine dimers (CPDs). In its absence, CPD lesions are bypassed by error‐prone TLS polymerases, leading to increased mutagenesis and heightened skin cancer susceptibility in XP‐V patients [19]. Nevertheless, recent studies revealed that XP‐V cells experience elevated oxidative stress following UVA irradiation, resulting in deleterious effects, including protein oxidation and reduced DNA repair capacity [20].

The mutagenic effects of UVA radiation in XP‐V cells have been investigated using next‐generation sequencing of clonal cell populations obtained from single irradiated XP‐V cells [21]. The predominant mutations identified included C > T transitions, C > A transversions, and CC > TT tandem mutations when cells were exposed to 120 kJ/m² of UVA, a dose relevant to tropical environments [22]. C > T transitions, particularly at dipyrimidine sites, indicate that CPDs are the primary lesion responsible for mutagenesis. Similarly, tumors from XP‐V patients also display a high frequency of C > A and C > T mutations [23, 24].

Despite these findings, questions remain regarding the mechanisms underlying UVA‐induced mutagenesis in XP cells [25], particularly those with clinical implications for patients [26]. Since UVA accounts for approximately 95% of the UV radiation that reaches the Earth's surface, further investigation is necessary. The reduction in pyrimidine dimer repair capacity following UVA exposure, as reported previously [20], highlights the critical role of oxidative stress in XP‐V cells, as the lower capacity of DNA damage removal may impact carcinogenesis in XP‐V patients.

Importantly, Moreno et al. [20] demonstrated that the antioxidant *N*‐acetylcysteine (NAC) offers protection to XP‐V cells by restoring their ability to repair pyrimidine dimers. NAC functions as a protective agent against excessive production of reactive oxygen species (ROS) by increasing intracellular cysteine availability for glutathione synthesis, while directly acting as an antioxidant [27]. Glutathione plays a vital role in protecting from oxidatively generated damage in mammalian cells [28]. This in vitro treatment with NAC holds a significant promise for developing strategies to reduce the impact of sunlight and improve the quality of life for XP‐V patients.

In this study, we sequenced the exomes of 28 XP‐V fibroblast clones and 24 clones of an isogenic line complemented with functional pol eta. The cells were treated with NAC (10 mM) before irradiation with 120 kJ/m² of UVA. NAC treatment provided significant protection for XP‐V cells, resulting in a ~ 57% reduction in exclusive point mutations compared to XP‐V cells exposed to UVA without NAC. This reduction was particularly pronounced in C > A transversions and C > T transitions, suggesting that NAC not only protects against nitrogenous base oxidation but also reduces mutations directly induced by UVA, likely arising from CPD lesions. These findings further underscore the critical role of oxidative stress in the absence of pol eta, which affects both translesion synthesis and DNA damage removal by NER.

## 2 Material and Methods

### 2.1 Cell Culture

Two strains of SV40‐transformed human fibroblasts derived from a XP‐V patient (XP30RO) [29] were used in this study: one strain is deficient in TLS (XP‐V) and the other proficient due to the complementation with the *POLH/XPV* gene (XP‐V_c) (XP30RO complemented—clone 6, gently provided by Drs. Anne Stary and Patricia Kannouche, Institut Gustave Roussy, Villejuif, France) [30]. The cells were cultured in Dulbecco's Modified Eagle Medium High Glucose—DMEM (LGC Biotechnologies, Cotia, SP, Brazil), supplemented with 10% heat‐inactivated fetal bovine serum (FBS) (Life Technologies, Carlsbad, CA, USA) and 1% antibiotic solution (0.1 mg/mL penicillin, 0.1 mg/mL streptomycin, and 0.25 mg/mL fungizone, Life Technologies). The cultures were maintained in a humidified atmosphere with 5% CO_2_ at 37°C.

### 2.2 Cloning and Extraction of Genomic DNA

The XP‐V and XP‐V complemented fibroblasts were plated at low density: 1–3 × 10^3^ cells per 100 mm dish for NAC treatment and UVA irradiation. Ten to fifteen days later, colonies were randomly collected and transferred into 24‐well plates. This transfer was performed manually using a 200 µL micropipette aided by the EVOS XL Core Imaging System microscope (ThermoFisher, Waltham, MA, USA). Approximately 30 days after the cell culture expansion, the genomic DNA was extracted with one of the following kits: QIAGEN Blood&CellCulture DNA MiniKit (Qiagen, Hilden, Nordrhein‐Westfalen, Germany) or MN NucleoSpin Tissue, Mini kit for DNA from cells and tissue (Dueren, North Rhine‐Westphalia, Germany).

### 2.3 Treatment With *N*‐Acetylcysteine and UVA Irradiation

The cells were treated with 10 mM of NAC (Sigma Aldrich, St. Louis, MO, USA) after adherence to the plate, approximately 4 h post‐plating. On the following day, from 16 to 20 h later, the culture medium was removed, and the plate was gently washed twice with phosphate‐buffered saline (PBS), before UVA irradiation in the presence of 10 mL of PBS. An Osram Ultramed FDA KY10s 1000 W UVA lamp associated with a 3 mm thick Schott BG39 filter (Schott Glass, Mainz, Rheinland‐Pfalz, Germany) was used, thus eliminating wavelengths below 320 nm and preventing the cells from receiving UVB and UVC radiation. The emission intensity of the lamp was measured before each experiment using the VLX 3 W radiometer (Vilber Lourmat, Torcy, France), and the exposure time was adjusted so that the cells received a total of 120 kJ/m^2^ of UVA radiation [20], while controls were kept in the dark with PBS.

### 2.4 Whole Exome Analysis Workflow

The complete exome sequencing was carried out by the Human Genome and Stem Cell Research Center (University of São Paulo, São Paulo, Brazil), using 5 μg of genomic DNA. For library preparation, either the Illumina Nextera Rapid Capture Custom Enrichment kit or DNA Prep kit (formerly known as Nextera DNA Flex, Illumina, San Diego, California, USA) was employed, and exome capture was performed using either the X‐Gen Exome Panel, version 1, or version 2 (IDT, Coralville, Iowa, USA), following the manufacturer's instructions. Paired‐end sequencing (2 × 150 bp) was carried out using the Illumina HiSeq. 2500 or NovaSeq. 6000 platforms.

Quality control of the raw data (FASTQ files) was conducted using FastQC (version 0.12.1) [31] and MultiQC (version 1.14) [32] tools. We employed the protocol established by the Broad Institute (MIT and Harvard, Massachusetts, USA), Genome Analysis Toolkit (GATK, version 4.2.0) for data preprocessing and variant calling [33, 34]. For mapping to the Grch38/Hg38 (https://console.cloud.google.com/storage/browser/gcp-public-data--broad-references) reference genome, the Burrows–Wheeler Aligner (BWA‐mem, version 0.7.17) [35] was used without altering the default parameters. The duplicate reads originating from the same DNA fragment were filtered using MarkDuplicates‐Picard tool (version 2.25.3), and finally base quality score recalibration (BQSR‐GATK) was performed before proceeding with variant calling using HaplotypeCaller (GATK). Variants exclusive to each clone were selected to avoid any bias related to germline mutations and cell culture handling. For this purpose, the BCFtools isec (version 1.12) tool [36] was used with the parameter ‐C (‐‐complement). Genes were annotated using ANNOVAR (version 2020‐06‐07) [37] with the RefSeq database [38], and finally, only variants found in exonic regions or splicing sites were selected. The main characteristics of the exome sequencing (sequencing platform, coverage, and number of bases read) are summarized in Supporting Information S1: Table S1.

### 2.5 Variant Analysis

An Initial exploratory analysis to identify point mutations was conducted using the WOLAND tool (https://github.com/tiagoantonio/woland/). Based on the six possible base substitutions (C:G > A:T, C:G > G:C, C:G > T:A, T:A > A:T, T:A > C:G, and T:A > G:C), WOLAND searches for variants in both transcribed and non‐transcribed strands. By obtaining the general patterns of single nucleotide variants (SNVs), it was possible to analyze their association with established motifs, such as pyrimidine dimers (YY, where Y corresponds to the pyrimidines: cytosine or thymine).

The analysis of somatic variant spectrum and mutational signatures induced by the mutagenesis process in the exome was performed using the Bioconductor [39] package SomaticSignatures (version 2.40.0) [40]. This package analyses the frequency of SNVs found within a trinucleotide context, considering the bases adjacent to positions 3′ and 5′ of the mutation site. To identify characteristic patterns of somatic mutations, the MutationalPatterns package (version 3.14) was applied [41]. This Bioconductor package was employed to determine the contribution of known mutational signatures cataloged in the Catalogue of Somatic Mutations in Cancer (COSMIC, version 3.4–October 2023). The contribution of these signatures was quantified using the refitting method, that mathematically optimizes the fitness of the signatures to the data. To minimize overfitting, the “backwards” and “best_subset” selection approaches were applied within the <fit_to_signatures_strict> function. The same package was used to analyze the transcriptional bias of point mutations. Transcript annotations from the UCSC Genome Browser for the Hg38 genome and the RefGene table, which contains RefSeq transcripts aligned by UCSC, were utilized for this analysis. According to the UCSC documentation, 2136 genes were excluded from the data set because they contain exons located on both strands of the same reference sequence or on multiple reference sequences, making them unsuitable for representation by a single genomic range.

To evaluate the sequence context within each mutation was inserted, considering three bases at 5′ position and other three at 3′ of the mutation, we used the Probability Logo Generator (pLogo, version 1.2.0) [42]. This tool generates the sequence context around the mutation based on binomial probability, enabling the inference of motif sequences. The enoLOGOS [43] was used to visualize the frequency of each nucleotide occurring around the mutation. Finally, the non‐negative matrix factorization (NMF) decomposition algorithm implemented in the SomaticSignatures enabled us to search for specific mutational patterns within the sequenced clones, thus allowing the inference of potential de novo mutational signatures while checking the cosine similarity between the patterns seen in our samples and known signatures from COSMIC database.

### 2.6 Statistical Analysis

To assess the significance of SNVs counts across different experimental conditions, pairwise permutation tests were performed between groups and treatments, with SNVs counts normalized to sequencing depth (40×). For each pairwise comparison, 5,000,000 permutations were conducted to compute *p* values. The robustness of the findings was assessed through sensitivity analyses, including variations in permutation counts and significance thresholds. Statistical significance for the one‐sided tests was defined as follows: * for *p* < 0.05, ** for *p* < 0.01, *** for *p* < 0.001 and ns (not significant) for *p* ≥ 0.05. *p* values were not adjusted for multiple testing. All analyses were conducted in R (version 4.4.1).

## 3 Results

### 3.1 Point Mutations: Initial Analysis

**Figure 1:**
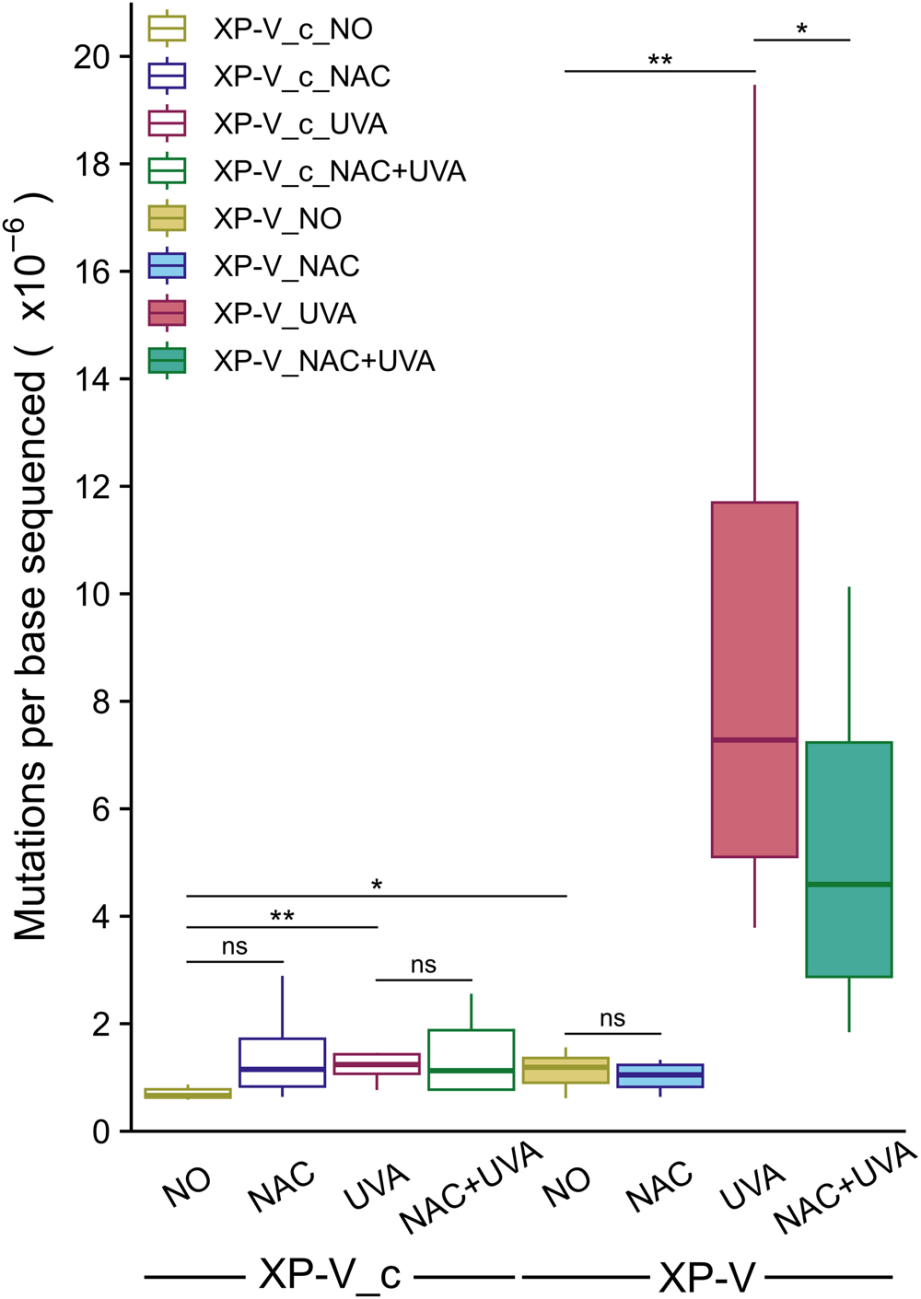
NAC reduced the number of SNVs induced by UVA radiation in XP‐V cells. Mutation counts were normalized by bases sequenced for each condition. XP‐V complemented (XP‐V_c) cells exhibited a significant increase in mutagenesis when irradiated with UVA (*p* = 0.0076). XP‐V cells showed a significant increase in mutagenesis upon UVA irradiation (*p* = 0.0011), which was significantly reduced when treated with NAC before irradiation (*p* = 0.0296). Additionally, the XP‐V strain accumulated more mutations than the XP‐V_c in the absence of UVA irradiation (*p* = 0.0109). *p* values were calculated using one‐sided pairwise permutation tests and were not adjusted for multiple testing. Statistical significance was defined as follows: * for *p* < 0.05, ** for *p* < 0.01, and ns (not significant) for *p* ≥ 0.05.

The absolute count of exclusive point mutations detected in untreated XP‐V_c (XP‐V_c NO) clones was low (165). XP‐V_c clones subjected only to NAC treatment (XP‐V_c NAC), only to UVA irradiation (XP‐V_c UVA), or combined NAC treatment followed by UVA irradiation (XP‐V_c NAC + UVA), exhibited nearly identical counts of SNVs (331, 371, and 327, respectively) (Table 1). No significant differences in InDels frequency were observed across different strains and treatments under any condition (Table 1). In contrast, UVA‐irradiated XP‐V clones (XP‐V UVA) accumulated mutations (SNVs + InDels) at a rate of 6.5 times higher than nonirradiated XP‐V cells (XP‐V NO), although pre‐treating cells with NAC before UVA irradiation (XP‐V NAC + UVA) reduced this ratio to 3.7. Overall, NAC treatment attenuated the increase in the count of mutations (SNVs + InDels) in UVA‐irradiated XP‐V clones by approximately 57% (Table 1 and Figure 1).

**Table 1:** Absolute count of mutations, separated into SNVs and InDels, identified by clone group. The columns expressed as percentages (%) were calculated based on the total count (SNVs + InDels). NO: untreated; NAC: 10 mM NAC; UVA: 120 kJ/m² UVA; NAC + UVA: 10 mM NAC and 120 kJ/m² UVA.

Groups	Clones (n)	SNVs (%)	InDels (%)	Total (normalized)^a^
XP‐V_c NO	6	165 (79.7)	42 (20.3)	207 (34.5)
XP‐V_c NAC	6	331 (80.9)	78 (19.1)	409 (68.2)
XP‐V_c UVA	6	371 (92.5)	30 (7.5)	401 (66.8)
XP‐V_c NAC + UVA	6	327 (88.9)	41 (11.1)	368 (61.3)
XP‐V NO	6	265 (76.1)	83 (23.9)	348 (58.0)
XP‐V NAC	6	239 (76.8)	72 (23.2)	311 (51.8)
XP‐V UVA	6	2164 (96.3)	84 (3.7)	2248 (374.7)
XP‐V NAC + UVA	10	2031 (94.5)	119 (5.5)	2150 (215.0)

a Total normalized by the number of clones of each group.

**Figure 2:**
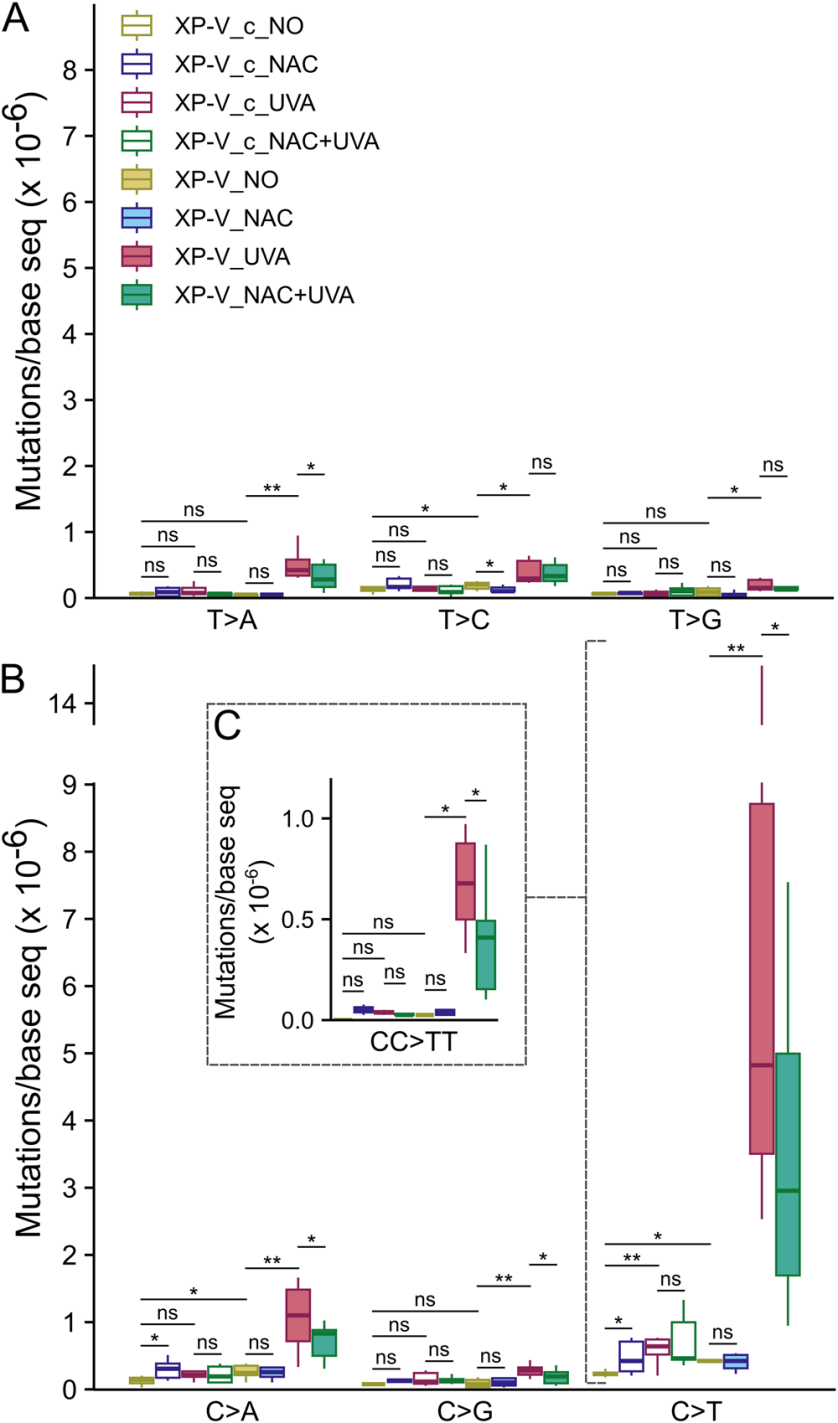
NAC specifically reduced C > A, C > G, C > T, and T > A point mutations induced by UVA radiation in XP‐V cells. Mutation counts were normalized by bases sequenced for each condition. (A) T > A, T > C, and T > G point mutations. UVA irradiation significantly increased the number of mutations in XP‐V cells for T > A (*p* = 0.0011), T > C (*p* = 0.0173), and T > G (*p* = 0.0431). NAC treatment before UVA irradiation reduced T > A mutations (*p* = 0.0406). A significant increase in T > C mutations was observed in XP‐V cells compared to XP‐V_c (*p* = 0.0281), which was reduced by NAC treatment in XP‐V cells without UVA irradiation (*p* = 0.0325). (B) C > A, C > G, and C > T point mutations. UVA irradiation significantly increased mutations in XP‐V cells compared to the nonirradiated condition (*p* = 0.0086, *p* = 0.0054, *p* = 0.0011 for C > A, C > G, and C > T, respectively). NAC treatment before UVA irradiation significantly reduced these mutations in XP‐V cells (*p* = 0.0341, *p* = 0.0136, and *p* = 0.0283 for C > A, C > G, and C > T, respectively). XP‐V cells also showed an increase in C > A (*p* = 0.0130) and C > T (*p* = 0.0109) mutations compared to their XP‐V_c counterparts. *p* values were calculated using one‐sided pairwise permutation tests and were not adjusted for multiple testing. Statistical significance was defined as follows: * for *p* < 0.05, ** for *p* < 0.01, and ns (not significant) for *p* ≥ 0.05.

In XP‐V cells, no significant alteration in endogenous mutagenesis (XP_V NO) was observed after NAC treatment (XP_V NAC) (Figure 1), except for a slight (but significant) decrease in T > C transitions (Figure 2A). As expected, and previously reported [21], UVA irradiation (XP_V UVA) induced mutations across all possible point mutation types in XP‐V cells, with a marked accumulation of C > A and C > T mutations (Figure 2B). Pretreatment with NAC (XP_V NAC + UVA) reduced the overall mutation count (Figure 1) and significantly decreased the occurrence of T > A (Figure 2A), C > A, C > G, and C > T mutations (Figure 2B). NAC treatment did not reduce mutations in either strain compared to their nontreated counterparts (Figure 1); however, it led to a slight increase in C > A and C > T mutations in the XP‐V_c cells (XP‐V_c NAC) (Figure 2B). UVA irradiation (XP‐V_c UVA) significantly increased C > T transitions in the XP‐V_c cells, and NAC treatment before irradiation (XP‐V_c NAC + UVA) did not mitigate this effect (Figure 2B).

Double adjacent mutations among the identified SNVs were rare across all clone sets, not exceeding 5% in the pol eta‐complemented clones and 4% in the nonirradiated XP‐V clones (Table 2). In contrast, the XP‐V UVA and XP‐V NAC + UVA clones exhibited higher rates of double mutations: 9.6% and 10.5%, respectively (Table 2). Upon UVA radiation, CC > TT tandem mutation increased for XP‐V strain (Table 2 and Figure 2C). Notably, the NAC treatment significantly reduced CC > TT tandem mutations in XP‐V UVA irradiated clones when compared to XP‐V UVA irradiated clones (Figure 2C).

**Table 2:** Absolute count of double base substitutions for each clone group and the percentage (%) of these mutations relative to the total number of SNVs. Count of CC > TT tandem mutations and their normalized count. NO: untreated; NAC: 10 mM NAC; UVA: 120 kJ/m² UVA; NAC + UVA: 10 mM NAC and 120 kJ/m² UVA.

Groups	Count of double mutations (%)	CC > TT (normalized)^a^
XP‐V_c NO	4 (2.4)	0 (0.0)
XP‐V_c NAC	14 (4.2)	4 (0.7)
XP‐V_c UVA	10 (2.7)	3 (0.5)
XP‐V_c NAC + UVA	8 (2.4)	5 (0.8)
XP‐V NO	4 (1.5)	3 (0.5)
XP‐V NAC	9 (3.8)	6 (1.0)
XP‐V UVA	208 (9.6)	158 (26.3)
XP‐V NAC + UVA	214 (10.5)	152 (15.2)

a Values normalized by the number of clones of each group.

### 3.2 Sequence Context of C > A Transversions and C > T Transitions

**Figure 3:**
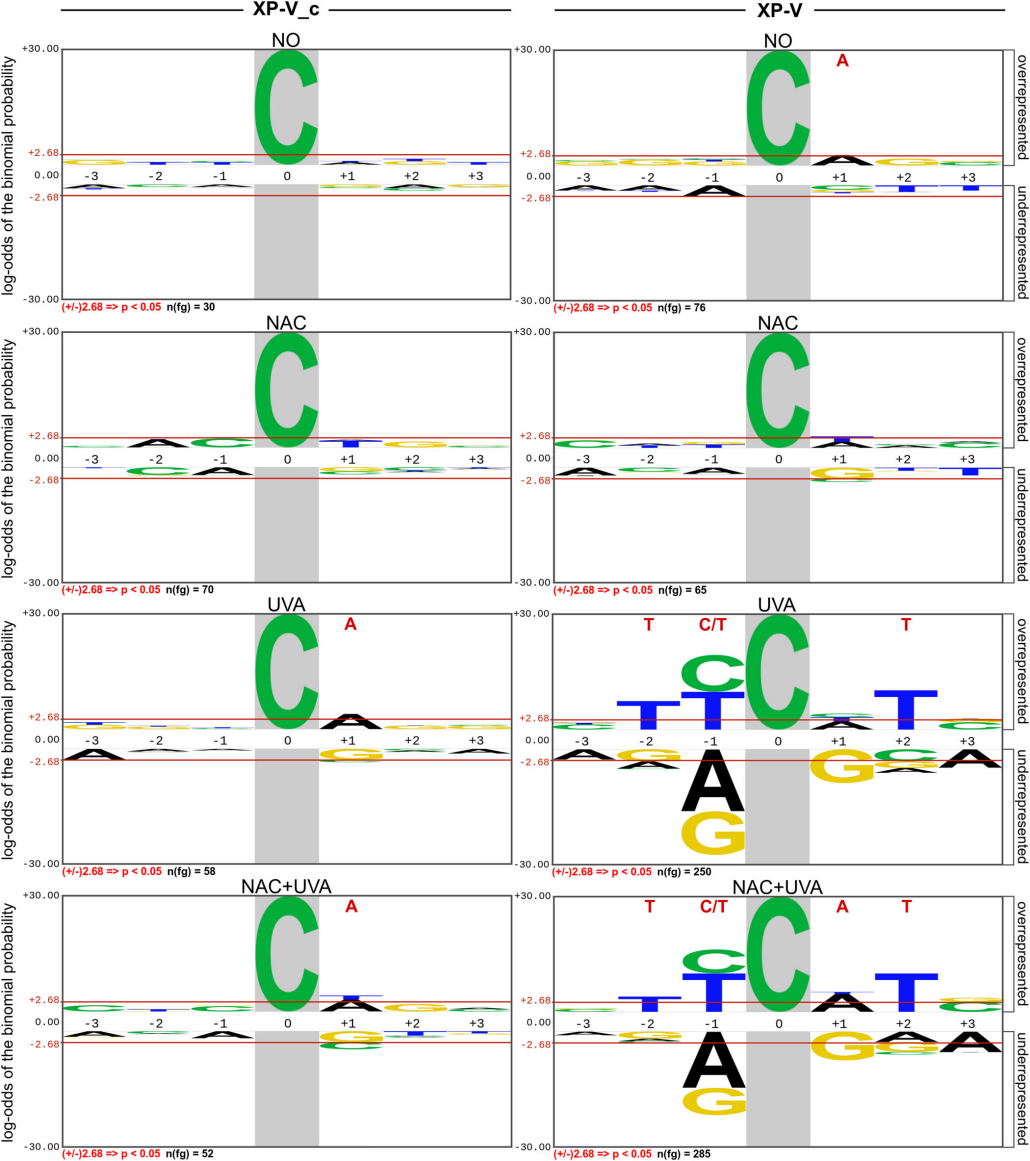
Sequence context surrounding C > A mutations detected across all conditions for XP‐V complemented and XP‐V cells. The Probability Logo Generator tool (pLogo, v1.2.0) was used to examine the sequence context adjacent to C > A mutations, which are highlighted with a gray background. The line positioned at 2.68 (binomial probability) corresponds to the corrected statistical significance based on the Bonferroni correction. Bases highlighted in red at the top of the logo are statistically significant. The y‐axis scale, representing the log‐odds of the binomial probability, was normalized across all logos. The human exome sequence was used as the background, and aligned sequences were used to construct the logo. For the background, 4096 sequences, with the foreground sequences (n(fg)) indicated beneath each condition.

The predominant point mutations observed in UVA‐irradiated XP‐V cells were C > A and C > T substitutions. To further investigate their sequence context, we performed an analysis with pLOGO. C > A transversions in irradiated XP‐V cells (XP‐V UVA) preferentially occurred at the third base of regions with pyrimidines at the 5′ end, following the TY **C** HT motif, where the bold‐underlined base represents the mutation site, and H denotes A, C, or T according to IUPAC nomenclature [44]. Notably, in cells pretreated with NAC before irradiation (XP‐V NAC + UVA), there was an enrichment not only of pyrimidines at the 5′ position of the mutated base but also of adenines followed by thymines at the 3′ end identified as the TY **C** AT motif (Figure 3).

Adenines were consistently enriched at the 3′ end of C > A mutations across all conditions in XP‐V cells, accounting for 40% in XP‐V NO (significant in Figure 3), 37% in XP‐V NAC, 32% in XP‐V UVA, and 36% in XP‐V NAC + UVA (Supporting Information S1: Figure S1). This observation aligns with previous studies, which reported a significant association of the **C** A motif with UVA‐irradiated XP‐V clones [21] and tumors from XP‐V patients [23]. Additionally, UVA‐irradiated XP‐V_c cells (XP‐V_c UVA and XP‐V_c NAC + UVA) also exhibited **C** A motifs associated with C > A mutations (Figure 3 and Supporting Information S1: Figure S1), supporting the role of UVA light in inducing these types of mutations.

**Figure 4:**
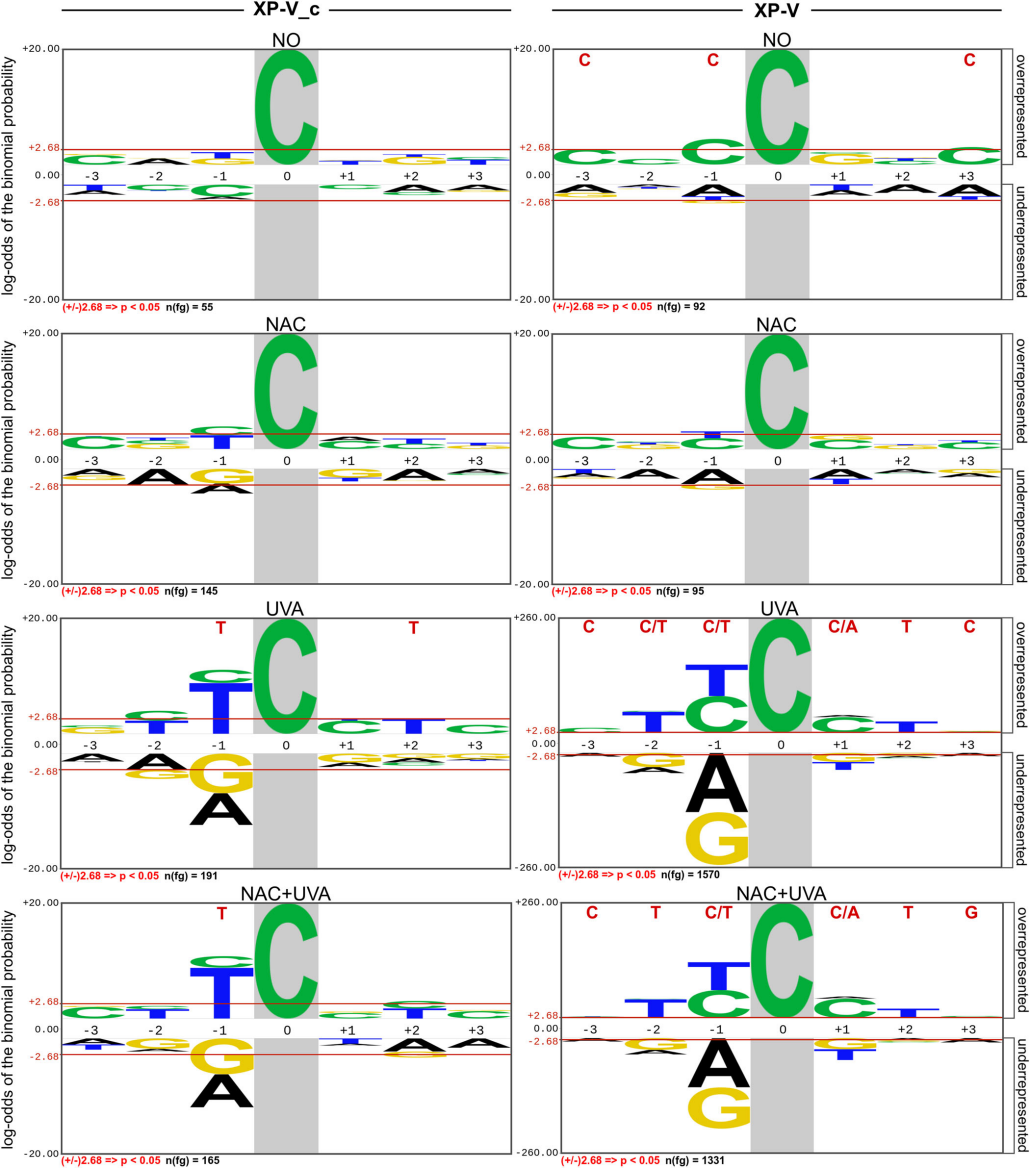
Sequence context surrounding C > T mutations detected across all conditions for the XP‐V complemented and XP‐V cells. The Probability Logo Generator tool (pLogo, v1.2.0) was used to examine the sequence context adjacent to C > T mutations, which are highlighted with a gray background. The line positioned at 2.68 (binomial probability) corresponds to the corrected statistical significance based on the Bonferroni correction. Bases highlighted in red at the top represent those with significant probability. The y‐axis scale, representing the log‐odds of the binomial probability, was normalized across all logos. Logos for XP‐V UVA and XP‐V UVA + NAC were normalized separately due to higher mutation accumulation in these conditions. The human exome sequence served as the background, and aligned sequences were used to construct the logo. For the background, 4096 sequences, with the foreground sequences (n(fg)) indicated beneath each condition.

C > T transitions were consistently the most frequent mutations observed across all conditions for both complemented and XP‐V cells (Figure 2B). The absolute count of C > T mutations occurring within pyrimidine dimer (YY) regions is presented in Table 3. In XP‐V cells exposed to UVA, approximately 96% of these mutations are localized in YY regions, regardless of NAC pretreatment, suggesting that C > T mutations specifically target pyrimidine dimer lesions. Sequence context analysis using pLOGO further emphasized the role of pyrimidine dimers as targets for UVA‐induced mutation. In both irradiated cell lines (XP‐V_c: UVA and NAC + UVA; XP‐V: UVA and NAC + UVA), pyrimidine‐rich contexts surrounding C > T mutations were evident (Figure 4 and Supporting Information S1: Figure S2). In XP‐V cells, C > T mutations were predominantly associated with the pyrimidine‐rich motifs CYY **C** MTC (where M denotes A or C, according to IUPAC nomenclature) for XP‐V UVA and CTY **C** MTG for XP‐V NAC + UVA. In contrast, UVA‐irradiated XP‐V_c cells displayed a simpler motif, T **C**, regardless of NAC pretreatment.

**Table 3:** Absolute count of C > T transitions and their percentage (%) relative to the total number of SNVs. Count of C > T transitions occurring in dipyrimidine regions (YY) and the percentage (%) of C > T transitions found in YY NO: untreated; NAC: 10 mM NAC; UVA: 120 kJ/m² UVA; NAC + UVA: 10 mM NAC and 120 kJ/m² UVA.

Groups	C > T (%)	YY	C > T in YY (%)
XP‐V_c NO	55 (33.3)	39	70.9
XP‐V_c NAC	145 (43.8)	112	77.2
XP‐V_c UVA	191 (51.5)	170	89.0
XP‐V_c NAC + UVA	165 (50.5)	142	86.1
XP‐V NO	92 (34.7)	68	73.9
XP‐V NAC	95 (39.7)	74	77.9
XP‐V UVA	1570 (72.6)	1514	96.4
XP‐V NAC + UVA	1331 (65.5)	1269	95.3

### 3.3 Trinucleotide Mutational Spectra Context, and Transcriptional DNA Strand Bias

**Figure 5:**
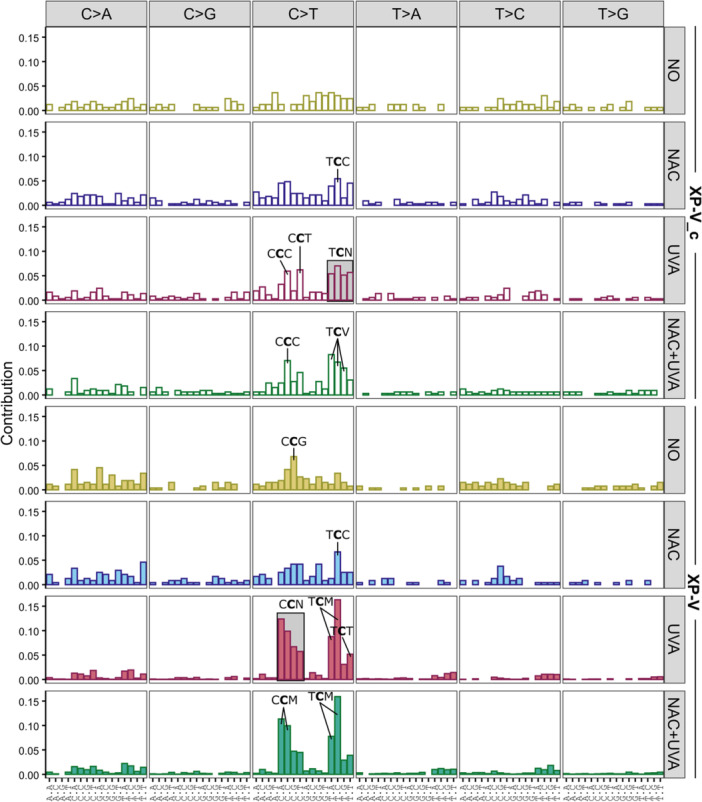
Somatic mutational spectra of XP‐V cells irradiated with UVA show a clear accumulation of mutations in dipyrimidine sites. The contribution of somatic spectra for all extracted point mutations is distributed in a trinucleotide context for complemented strain (XP‐V_c) (shown in lighter colors, first four rows) and XP‐V strain (shown in darker colors, last four rows), untreated (NO), treated with NAC, irradiated with UVA, and treated with NAC before UVA exposure (NAC + UVA). The contribution represents the mutation frequency for each type of point mutation for each clone set, categorized by lineage and treatment type. On the lower x‐axis, the dot should be replaced by the corresponding mutation defined on the upper x‐axis. Only point mutations with a contribution greater than or equal to 0.05 are highlighted. According to IUPAC, M is replaced by A or C, N is replaced by A, C, G, or T, and V is replaced by A, C, or G.

The mutational spectra illustrate the influence of immediately adjacent bases on mutation patterns by analyzing their frequency within a trinucleotide context. In Figure 5, only point mutations with a contribution equal to or greater than 0.05 are highlighted, all of which are exclusively C > T transitions.

Untreated XP‐V_c cells did not display any motifs identified as statistically significant (Figures 3 and 5). However, NAC treatment in these cells led to a modest increase in mutations within the T **C** C context. UVA irradiation resulted in a comparable increase in the count of mutations in XP‐V_c cells, irrespective of NAC treatment. Notably, the irradiation that followed the NAC treatment induced an enrichment of mutations in the C **C** C context, with a predominant accumulation in the T **C** V motif (where V represents A, C, or G) (Figure 5).

In the XP‐V deficient cells, the untreated group showed a modest increase in C **C** G trinucleotide mutations, while NAC treatment led to an enrichment in the T **C** C motif. C > T transitions were the most prevalent mutations observed across all conditions. However, only in UVA‐irradiated XP‐V cells did the mutational spectra display a distinct pattern, predominantly associated with mutations at dipyrimidine sites. This trend was consistent regardless of NAC pretreatment (Figure 5). Interestingly, UVA‐irradiated XP‐V cells accumulated T > A transversions at dipyrimidine sites (Supporting Information S1: Figure S3), specifically within the T **T** N context.

**Figure 6:**
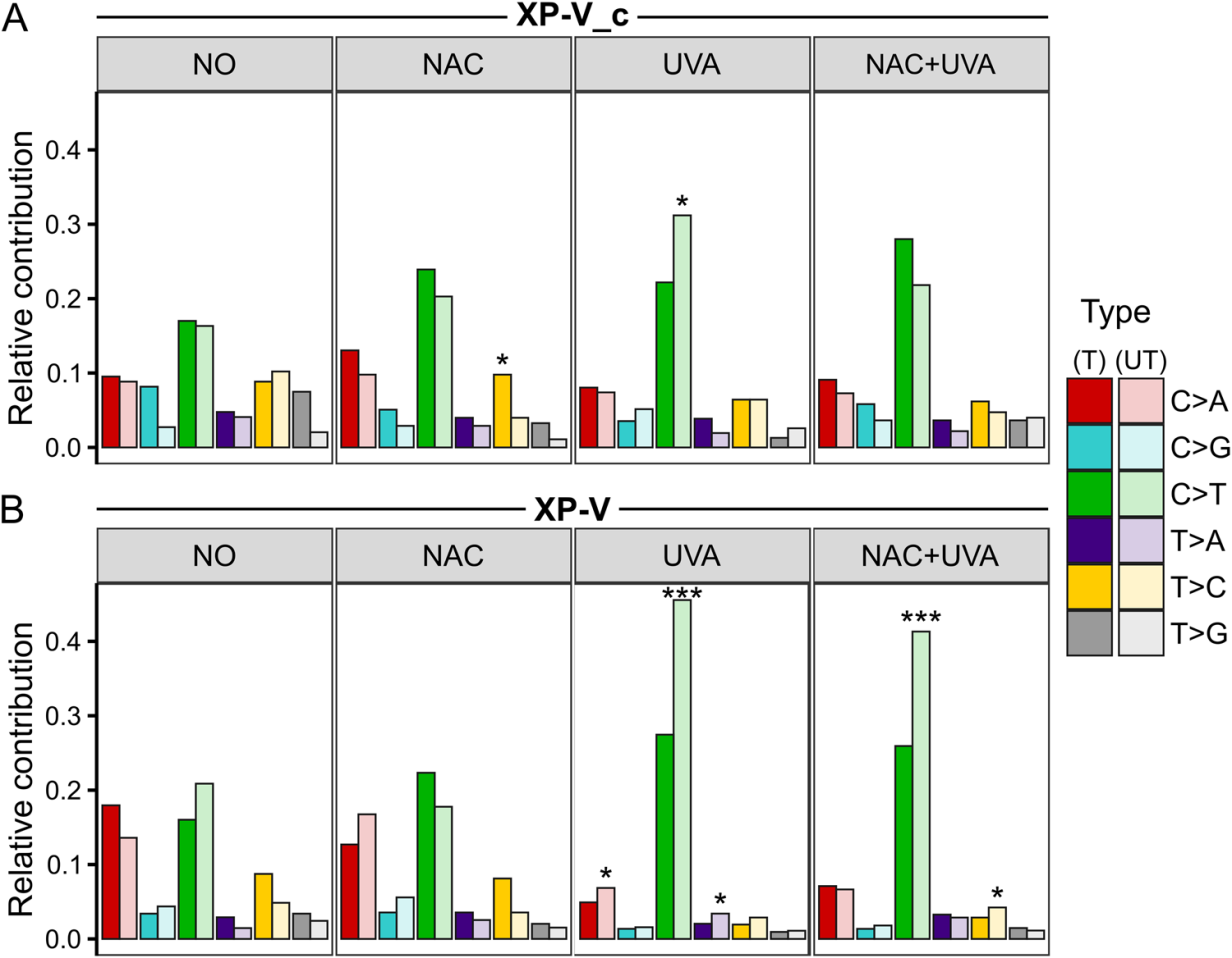
Strand‐specific analysis of point mutations: XP‐V cells predominantly accumulated mutations on the untranscribed strand after UVA irradiation. (A) In complemented cells (XP‐V_c) treated with NAC, T > C transitions were enriched on the transcribed strand (*p* = 0.0139), while UVA irradiation shifted this bias to C > T at the untranscribed strand (*p* = 0.0358). (B) UVA‐irradiated XP‐V cells showed significant accumulation of C > A (*p* = 0.0162), C > T (*p* = 1.68×10^−20^), and T > A (*p* = 0.0138) mutations on the untranscribed strand. NAC treatment before UVA exposure eliminated the strand bias for C > A transversions, while the strand discrepancies persisted for C > T (*p* = 2.59×10^−^^15^) and introduced a minor bias for T > C (*p* = 0.0400). The vertical axis indicates the relative contribution of mutations on each strand, categorized by clone group and mutation type. Mutations on the coding (sense) strand are classified as Untranscribed (UT, lighter colors), and those on the opposite strand as Transcribed (T, darker colors). Mutations overlapping gene bodies on both strands were excluded from this analysis. Statistical significance: * ﻿﻿for *p* < 0.05, *** for *p* < 0.001.﻿﻿

For the transcriptional DNA strand bias analysis, base substitutions were categorized based on their location relative to the coding (sense) strand: those on the coding strand were designated as untranscribed (UT‐strand), while those on the complementary strand were classified as transcribed (T‐strand). Substitutions overlapping gene bodies on both strands were excluded from the strand‐specific analysis. Mutation counts (Supporting Information S1: Table S2) and the relative contributions of mutations on each strand were considered. A transcriptional bias was observed in XP‐V_c cells treated with NAC, with an enrichment of T > C transitions in the T‐strand and C > T transitions in the UT‐strand for irradiated cells (XP‐V_c UVA) (Figure 6A). In XP‐V cells, a strong bias of C > T transitions, and minor for C > A and T > A, toward the UT‐strand was identified exclusively in irradiated samples (Figure 6B). Treatment of XP‐V cells with NAC before UVA exposure (XP‐V NAC + UVA) effectively eliminated the C > A and T > A bias but kept the strong enrichment of C > T in the UT‐strand (Figure 6B).

### 3.4 Mutational Signature Fitting Using Cosmic Database

**Figure 7:**
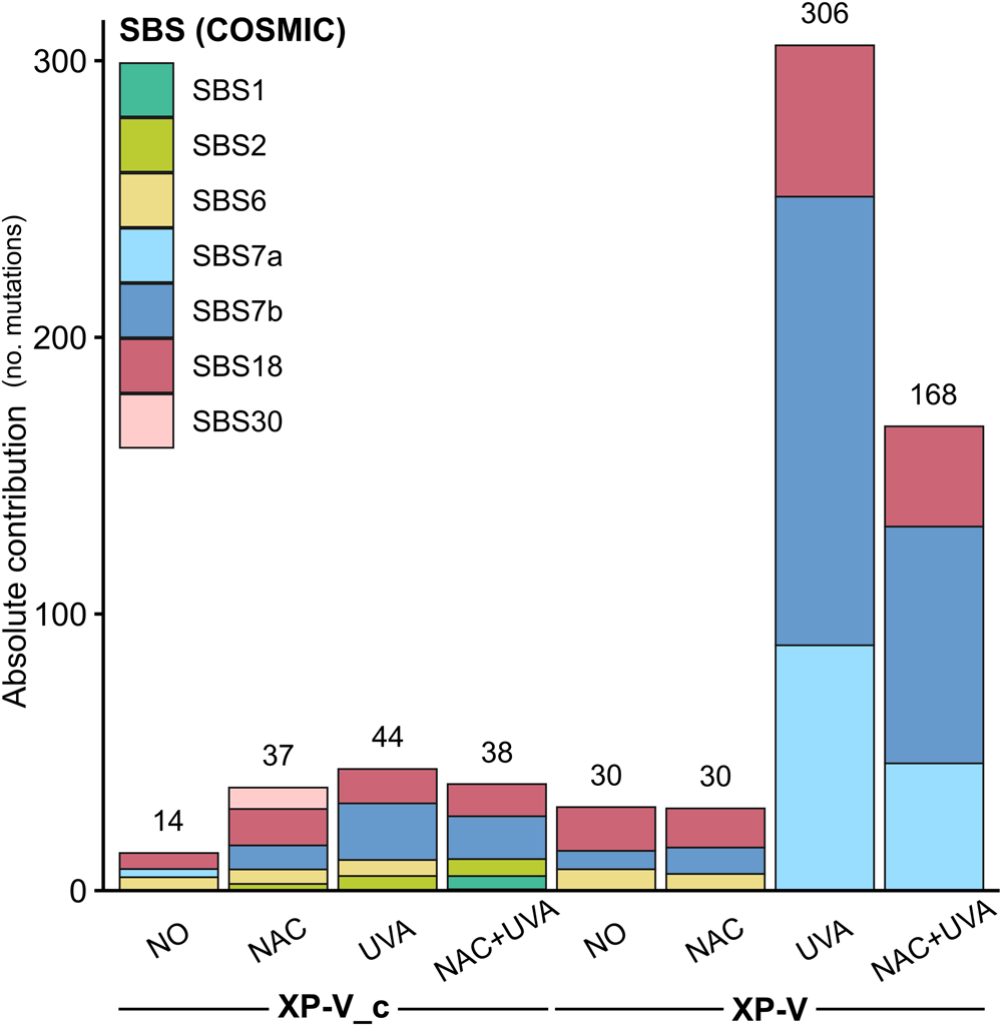
COSMIC Mutational signatures SBS7a and SBS7b were distinctly associated with point mutations identified for XP‐V cells irradiated with UVA. Contributions of single base substitution (SBS) signatures 1 (spontaneous deamination of 5‐methylcytosine (clock‐like signature), 2 (activity of the APOBEC family of cytidine deaminases), 6 (defective DNA mismatch repair), 7a and 7b (ultraviolet light exposure), 18 (damage by reactive oxygen species), and 30 (defective DNA base excision repair due to NTHL1 mutations), as cataloged in the COSMIC database. XP‐V UVA and XP‐V treated with NAC before UVA irradiation exhibited a higher contribution of signatures 7a and 7b (total of 82% and 78%, respectively), reflecting the direct DNA mutations induced by UVA radiation, which are characteristic of these signatures.

The single base substitutions (SBS) identified in each clone set were compared to mutational signatures cataloged in version 3.4 of the COSMIC database, which compiles experimentally validated mutational profiles associated with distinct mutagenic processes. The resulting mutational profiles are summarized in Figure 7. For XP‐V_c NO cells, approximately 50% of all identified SNVs matched COSMIC signatures, while ~70% of SNVs from the other conditions involving pol eta‐complemented clones aligned with these profiles. Similarly, 68% and 75% of the SNVs from XP‐V NO and XP‐V NAC cells, respectively, contributed to reconstructing their mutational signatures. For the XP‐V UVA and XP‐V NAC + UVA cells, 85% and 83% of the identified SNVs, respectively, were used to understand their mutational processes. These matches highlight the relevance of COSMIC mutational signatures in deciphering the underlying mechanisms of mutagenesis in these experimental conditions.

Untreated XP‐V_c cells displayed a similar contribution from the SBS6 and SBS18 mutational signatures, with a minor contribution from the SBS7a signature (Figure 7). The SBS6 signature is associated with DNA mismatch repair deficiencies and is frequently observed in tumors with microsatellite instability. The SBS18 signature, linked to damage induced by ROS, is characterized by a predominance of C > A transversions. In contrast, XP‐V_c cells treated with NAC exhibited contributions from SBS2, SBS6, SBS7b, SBS18, and SBS30 signatures. Mutations resembling the SBS2 signature are attributed to the activity of APOBEC enzymes, a family of cytidine deaminases, and may result from DNA replication across uracil or error‐prone polymerases synthesizing DNA at abasic sites generated during the BER‐mediated removal of uracil. The SBS30 signature is associated with inactivation of the *NTHL1* gene, which encodes a DNA glycosylase that removes oxidized pyrimidines [45].

Notably, the contributions of COSMIC mutational signatures to XP‐V NO and XP‐V NAC clones were comparable (Figure 7), as both clones accumulated a similar number of point mutations and exhibited analogous mutational profiles. Signatures associated with UV light (SBS7a and SBS7b) were predominant in cells exposed to UVA radiation. As expected, irradiated XP‐V cells, which exhibited a high level of mutagenesis, showed a pronounced contribution from these signatures (Figure 7). The strong association of SBS7a and SBS7b with UV‐induced lesions, primarily pyrimidine dimers, further supports previous findings [21]. Only XP‐V_c cells treated with NAC before UVA (XP‐V_c NAC + UVA) irradiation exhibited a minor contribution from SBS1, associated with an endogenous mutational process characterized by the spontaneous or enzymatic deamination of 5‐methylcytosine to thymine. Finally, both XP‐V_c UVA and XP‐V_c NAC + UVA showed a higher contribution of SBS7b, which is associated with UVA‐induced damage, occurring even in cells with functional pol eta and intact repair mechanisms.

Despite the significant reduction in mutation frequency observed with NAC treatment in UVA‐irradiated XP‐V cells (Figure 7), the mutational signature profile remained largely unchanged (Figure 5). This suggests that while NAC effectively mitigates the extent of mutagenesis, it does not alter the type of DNA lesion responsible for mutations.

### 3.5 De Novo Mutational Signatures Extraction and Fitting

Mutational signatures for each cell line and treatment were inferred based on the frequency of somatic motifs using the non‐negative matrix factorization (NMF) algorithm [46], implemented in the SomaticSignatures package. Application of NMF to the matrix of SNVs identified revealed five mutational signatures (S1, S2, S3, S4, and S5), collectively accounting for 97% of the observed data (Supporting Information S1: Figure S4A).

Signature S1, identified as SBS7b‐like (cosine similarity with SBS7b from COSMIC (CS) > 0.87), was predominantly associated with the XP‐V UVA and XP‐V NAC + UVA clone sets, contributing 98% and 83%, respectively (Supporting Information S1: Figure S4B). These cells displayed a high degree of similarity to S1 (CS > 0.99) and to each other (Supporting Information S1: Figures S4C and S4D).

The XP‐V_c cells treated with NAC, irradiated, or subjected to both NAC treatment and irradiation (XP‐V_c NAC, XP‐V_c UVA, and XP‐V_c NAC + UVA) displayed contributions of 30%, 100%, and 52%, respectively, from signature S2 ((Supporting Information S1: Figure S4B). Their mutational spectra showed considerable similarity to S2 (CS: 0.87, 0.98, and 0.94) (Supporting Information S1: Figure S4C) and to each other, with CS values of 0.85 for XP‐V_c NAC versus XP‐V_c UVA, 0.84 for XP‐V_c NAC versus XP‐V_c NAC + UVA, and 0.89 for XP‐V_c UVA versus XP‐V_c NAC + UVA (Supporting Information S1: Figure S4D). Signatures S3 and S4 closely reconstructed the mutational spectra of the XP‐V NAC and XP‐V NO cells, with each contributing 100% (Supporting Information S1: Figure S4B) and exhibiting high similarity to their respective signatures (CS > 0.99) (Supporting Information S1: Figure S4C). The untreated XP‐V complemented cells (XP‐V_c NO) aligned with signature S5, showing a similarity of 0.99 (Supporting Information S1: Figure S4C) and a 100% contribution (Supporting Information S1: Figure S4B).

## 4 Discussion

This study aimed to evaluate whether the antioxidant *N*‐acetylcysteine (NAC) could relieve the mutagenic effects of UVA radiation in XP‐V cells. NAC acts as an antioxidant that increases intracellular cysteine levels (a precursor of glutathione), thus helping to control oxidative stress induced by UVA exposure [20, 27]. Additionally, NAC partially prevents the loss of capacity of XP‐V cells to repair DNA damage through mechanisms associated with base excision repair (BER) and nucleotide excision repair (NER), ultimately enhancing cell viability and reducing cell death in vitro [20].

The results revealed a significant increase in all six possible types of single nucleotide mutations in XP‐V cells exposed to UVA radiation compared to isogenic cells complemented with polymerase eta. Consistent with previous findings [21], C > T transitions and C > A transversions emerged as the most frequent mutations induced by UVA irradiation in XP‐V cells. UVA radiation is well known to directly induce DNA lesions, including CPDs, and to cause indirect damage through the oxidation of nitrogenous bases in the DNA molecule [8, 9, 47]. Interestingly, pretreatment with NAC before UVA exposure significantly reduced C > T, C > A, C > G, and T > A point mutations in XP‐V cells. However, T > C and T > G mutations were less frequent and unaffected by NAC treatment, suggesting that oxidative stress is not involved in the induction of these types of mutation by UVA radiation (Figure 2).

Numerous studies have shown that pol eta enables error‐free bypass of CPDs during replication by accurately incorporating bases opposite these dimers on the template strand [19]. The present study focused on the significance of mutations in dipyrimidine contexts within pol eta deficient XP‐V cells. Notably, mutations involving thymine (T) are rare in these cells, despite TT dimers being the most common lesions induced by UVA radiation [48]. Consistent with previous findings [21], the mutations detected in thymine predominantly occurred at dipyrimidine sites (Supporting Information S1: Figure S3). This pattern is particularly evident for T > A transversions, which were primarily observed in T **T** N contexts. These observations strongly implicate TT CPDs as the primary targets of T > A transversions in the absence of functional pol eta.

In XP‐V cells, pol iota acts as the primary TLS polymerase that compensates for the absence of pol eta during CPD bypass [49, 50]. Previous studies have demonstrated that pol iota tends to be error‐prone during TT CPD bypass, frequently incorporating thymine on the opposite strand [51]. This error‐prone mechanism likely contributes to the emergence of UVA‐induced T > A transversions, in the absence of pol eta.

The second most frequent type of mutation identified in UVA‐irradiated XP‐V cells was C > A (G > T) transversions, which are often associated with the error‐prone replication of 8‐oxo‐7,8‐dihydroguanine (8‐oxoG) in DNA, a byproduct of oxidative stress. 8‐oxoG has been identified as the primary type of DNA damage caused by UVA radiation through endogenous photosensitizers and the production of singlet oxygen, which reacts with the guanine base [52, 53]. Additionally, UVA irradiation of DNA can generate singlet oxygen, leading to the formation of 8‐oxoG in the molecule [54]. Treatment with NAC significantly reduced the accumulation of this mutation type, although the sequence context and mutation spectra remained similar in both conditions (TY **C** NT for XP‐V UVA and TY **C** AT for XP‐V NAC + UVA) (Figure 3). Interestingly, UVA irradiation led to an accumulation of C > A mutations predominantly on the untranscribed strand in XP‐V cells, probably a consequence of transcription‐coupled nucleotide excision repair (TC‐NER), a sub‐pathway of NER that repairs DNA damage in the transcribed strand of active genes [55]. In contrast, XP‐V cells treated with NAC before UVA irradiation exhibited no strand‐specific bias for C > A mutations, suggesting that the antioxidant effectively diminished the overall occurrence of these mutations (Figure 6). As pol eta was reported to also act on the bypass of 8‐oxoG [56], the increased frequency of C > A mutations in UVA‐irradiated XP‐V cells, indicates that pol eta may also play a protective role against 8‐oxoG‐induced mutagenesis. Alternatively, the changes might enhance the repair capacity for 8‐oxoG or other lesions responsible for this type of mutation. Importantly, C > A transversions are frequently observed in XP‐V tumors, representing a unique mutational signature associated with XP‐V cells and underscores their critical relevance to the XP‐V patient phenotype [23, 24].

C > T transitions accounted for 73% of all point mutations observed in UVA‐irradiated XP‐V cells. Pretreatment with NAC significantly reduced these mutations, though C > T transitions still represented 66% of the mutations induced by UVA irradiation. In both cases, mutations occurred predominantly in dipyrimidine sites, particularly in pyrimidine‐rich regions (Figure 4). The mutational spectra induced by UVA irradiation in XP‐V cells (Figure 5), when examined within a trinucleotide context, showed that C > T transitions were concentrated at dipyrimidine sites, especially when a pyrimidine was located at 5′ to the mutation. Regardless of NAC pretreatment, UVA irradiation in XP‐V cells predominantly induced mutations associated with the mutational signature S1, which closely resembles the COSMIC SBS7b signature. The COSMIC SBS7a and SBS7b signatures, which also accumulate C > T mutations at the untranscribed strand, accounted for most of the mutations in UVA‐irradiated XP‐V cells, irrespective of NAC pretreatment (Figure 7). Furthermore, a significant proportion of the C > T mutations detected in these cells were tandem CC > TT mutations, a hallmark of UV‐induced mutations characteristic of this UV light spectrum [57].

These results confirm that C > T mutations in UVA‐irradiated XP‐V cells exhibit similar profiles, independent of the NAC pretreatment. It is likely that these mutations arise from the erroneous replication of CPDs in the absence of functional pol eta, as observed in UVA‐irradiated XP‐V cells and in XP‐V tumors [20, 23, 24]. Earlier studies have shown that UVA irradiation induces oxidative stress in XP‐V cells and inhibits NER [20], confirming that protein oxidation by UVA light in different conditions inhibits NER [58, 59]. The negative impact of UVA‐light on the removal of CPDs was also observed in other cell models, including human keratinocytes [60] and primary human melanocytes [61]. Interestingly, recent data in human reconstructed epidermis revealed that even visible light can inhibit the repair of CPDs induced by a subsequent UVB irradiation [62]. Therefore, the reduced efficiency of CPD removal by NER in UVA‐irradiated XP‐V cells may partially explain the increased mutagenesis. The decrease in mutation frequency, particularly at dipyrimidine sites, following NAC treatment supports the hypothesis that NAC protects NER proteins from oxidative stress, thereby enhancing CPD removal [20].

Since C > T mutations caused by pyrimidine dimers are the most commonly detected alterations in tumors from XP‐V patients, the impaired capacity to repair CPDs—due to the oxidation of NER‐related proteins—may contribute substantially to the elevated mutational burden observed in these cells and, consequently, to the increased incidence of skin tumors in affected individuals. This mechanism explains why at least part of the XP‐V clinical phenotype shares features with XP‐C, a classical NER‐deficient form of the disease, despite the later onset and typically milder tumor progression observed in XP‐V patients.

Surprisingly, NAC treatment in XP‐V complemented cells (XP‐V_c_NAC) led to a significant increase in mutations, compared to untreated controls (XP‐V_c_NO) and even matching those seen in irradiated groups. Notably, C > A and C > T mutations also increased, and no protective effect was detected. These unexpected results, in light of NAC's reported anti‐ and pro‐carcinogenic effects [63, 64, 65], underscore the need for further investigation to clarify whether NAC might induce mutagenesis in cells with functional pol eta, potentially by disrupting the balance of ROS metabolism. Another interesting aspect is that antioxidants can only scavenge low‐reactive oxygen species, including the superoxide anion radical and, to a lesser extent, singlet oxygen [66]; thus, the use of NAC should alter the DNA lesions and mutation profiles induced by UVA. However, in this study, the use of NAC resulted in a reduction in the frequency of mutation, but there was no significant change in the mutation profile induced by UVA. Moreover, determining the mutagenicity by direct specific oxygen species (such as singlet oxygen, induced by UVA with photosensitizers) could help in understanding the roles of pol eta in the protection against specific DNA lesions (such as 8‐oxoG).

In conclusion, this study highlights the critical role of UVA‐induced oxidative stress in compromising DNA repair efficiency and promoting mutagenesis in XP‐V cells. Our findings emphasize that the increase in the frequency of mutations in these patients arises not solely from TLS deficiency, but also from impaired NER function due to oxidative protein damage. Importantly, antioxidant intervention with NAC demonstrated a dual protective effect by reducing C > A transversions, through reduction of oxidative stress, and lowering C > T transitions, likely by preventing oxidatively generated damage to NER proteins and partially restoring NER activity. While topical NAC poses limitations due to its potential to act as a chromophore and generate ROS upon UVA exposure, systemic antioxidant supplementation remains a promising alternative to protect XP‐V patients. Given that UVA constitutes approximately 95% of solar UV radiation and that oxidative stress from intense sunlight can also impair DNA repair in the general population, reduced CPD clearance may contribute to skin tumorigenesis beyond XP‐V patients. Therefore, the integration of antioxidants into sunscreen formulations may hold promise as a complementary strategy to mitigate pyrimidine dimer‐induced DNA damage. Several commercial sunscreens already contain antioxidants, with vitamin E and its derivatives being the most frequently used, followed by vitamin C, ferulic acid, and niacinamide, all of which have demonstrated varying degrees of antioxidant and photoprotective effects in vivo and clinical studies, despite some inconsistencies in vitro [67]. However, it is crucial to note that further research is needed to determine their efficacy, specifically in XP‐V or similarly vulnerable individuals. The potential of combining antioxidants with traditional photoprotective agents to enhance protection against UVA‐induced skin damage is significant. This approach could support the development of targeted strategies to reduce mutagenesis in XP‐V patients and promote broader skin health in the general population.

## Author Contributions

**Camila Corradi:** conceptualization, methodology, experimental design and execution, data curation, statistical analysis, original draft preparation, review, and editing. **Natália Cestari Moreno:** supervision, methodology development, and critical manuscript review. **Nathalia Quintero‐Ruiz:** data validation, statistical analysis, critical review, and editing. **Giovana da Silva Leandro and Marcela Teatin Latancia:** critical review and editing of the manuscript. **Tiago Antonio de Souza:** software support. **Veridiana Munford:** resource provision and critical manuscript review. **Carlos Frederico Martins Menck:** conceptualization, supervision, funding acquisition, resource provision, critical review, and editing.

## Conflicts of Interest

The authors declare no conflicts of interest.

## Supporting information


**Supplementary Fig. S1:** Frequency of context sequences surrounding C > A mutations detected in all conditions of XP‐V complemented (XP‐V_c) and XP‐V cells. The enoLOGOS tool was used to examine the frequency of context sequences adjacent to C > A mutations, which are highlighted with a gray background. The human exome sequence served as the background, and the aligned sequences were used to construct the logo. NO: untreated; NAC: 10 mM NAC; UVA: 120 kJ/m^2^ UVA; NAC+UVA: 10 mM NAC and 120 kJ/m^2^ UVA. **Supplementary Fig. S2:** Frequency of context sequences surrounding C>T mutations detected in all conditions of XP‐V complemented (XP‐V_c) and XP‐V cells. The enoLOGOS tool was used to examine the frequency of context sequences adjacent to C > T mutations, which are highlighted with a gray background. The human exome sequence served as the background, and the aligned sequences were used to construct the logo. NO: untreated; NAC: 10 mM NAC; UVA: 120 kJ/m² UVA; NAC+UVA: 10 mM NAC and 120 kJ/m² UVA. **Supplementary Fig. S3:** T > A point mutations somatic spectra of XP‐V complemented and XP‐V cells in detail. The contribution of somatic spectra only for T > A point mutations distributed in a trinucleotide context for complemented strain (XP‐V_c) (shown in lighter colors, first four rows) and XP‐V strain (shown in darker colors, last four rows), untreated (NO), treated with NAC, irradiated with UVA, and treated with NAC prior to UVA exposure (NAC+UVA). The contribution represents the mutation frequency for each type of point mutation for each clone set, categorized by lineage and treatment type. On the lower x‐axis, the dot should be replaced by T>A mutation. According to IUPAC, N is replaced by A, C, G, or T. NO: untreated; NAC: 10 mM NAC; UVA: 120 kJ/m^2^ UVA; NAC+UVA: 10 mM NAC and 120 kJ/m^2^ UVA. **Supplementary Fig. S4:** Relative contribution of five mutational signatures reconstructed from point mutations across all conditions of XP‐V complemented (XP‐V_c) and XP‐V cells. (A) Composition of the mutational spectra for signatures S1, S2, S3, S4, and S5, reconstructed by non‐negative matrix factorization (NMF). Signature S1 shows a high cosine similarity (> 0.88) with the SBS7b signature (UV exposure) from COSMIC and is therefore identified as SBS7b‐like. (B) Graphical representation of the contribution of each reconstructed signature to the mutational spectrum of the clones. (C) Cosine similarity between the estimated signatures and the mutational profile of each clone set. (D) Cosine similarity between the mutational profiles of each clone set. NO: untreated; NAC: 10 mM NAC; UVA: 120 kJ/m^2^ UVA; NAC+UVA: 10 mM NAC and 120 kJ/m^2^ UVA. **Supplementary Table S1:** Depth of coverage and alignment metrics of XP‐V complemented (XP‐V_c) and XP‐V cells, grouped by treatment and no treatment conditions. NO: untreated; NAC: 10 mM NAC; UVA: 120 kJ/m^2^ UVA; NAC+UVA: 10 mM NAC and 120 kJ/m^2^ UVA. **Supplementary Table S2:** Absolute count of mutations on each strand, per group of clones and per mutations type. Base substitutions on the coding (sense) strand were classified as Untranscribed, while those on the opposite strand are Transcribed. Substitutions overlapping gene bodies on both strands were excluded from strand‐specific analysis. NO: untreated; NAC: 10 mM NAC; UVA: 120 kJ/m^2^ UVA; NAC+UVA: 10 mM NAC and 120 kJ/m^2^ UVA.

## Data Availability

Sequencing data that support the findings of this study are openly available in the National Center for Biotechnology Information Sequence Read Archive (SRA) under accession numbers PRJNA977685.
