# Supplementary material for: Uncovering the Role of DNA Repair Impairment in UVA‐Induced Mutagenesis in Human Xeroderma Pigmentosum Variant Cells
Source: Mol Carcinog. 2025 Aug 12;64(11):1823–37. doi: 10.1002/mc.70028 (PMC12535409; doi:10.1002/mc.70028)
Supplement: Supplementary file 1 — Supplementary Fig. S1: Frequency of context sequences surrounding C > A mutations detected in all conditions of XP‐V complemented (XP‐V_c) and XP‐V cells. The enoLOGOS tool was used to examine the frequency of context sequences adjacent to C > A mutations, which are highlighted with a gray background. The human exome sequence served as the background, and the aligned sequences were used to construct the logo. NO: untreated; NAC: 10 mM NAC; UVA: 120 kJ/m2 UVA; NAC+UVA: 10 mM NAC and 120 kJ/m2 UVA. Supplementary Fig. S2: Frequency of context sequences surrounding C>T mutations detected in all conditions of XP‐V complemented (XP‐V_c) and XP‐V cells. The enoLOGOS tool was used to examine the frequency of context sequences adjacent to C > T mutations, which are highlighted with a gray background. The human exome sequence served as the background, and the aligned sequences were used to construct the logo. NO: untreated; NAC: 10 mM NAC; UVA: 120 kJ/m² UVA; NAC+UVA: 10 mM NAC and 120 kJ/m² UVA. Supplementary Fig. S3: T > A point mutations somatic spectra of XP‐V complemented and XP‐V cells in detail. The contribution of somatic spectra only for T > A point mutations distributed in a trinucleotide context for complemented strain (XP‐V_c) (shown in lighter colors, first four rows) and XP‐V strain (shown in darker colors, last four rows), untreated (NO), treated with NAC, irradiated with UVA, and treated with NAC prior to UVA exposure (NAC+UVA). The contribution represents the mutation frequency for each type of point mutation for each clone set, categorized by lineage and treatment type. On the lower x‐axis, the dot should be replaced by T>A mutation. According to IUPAC, N is replaced by A, C, G, or T. NO: untreated; NAC: 10 mM NAC; UVA: 120 kJ/m2 UVA; NAC+UVA: 10 mM NAC and 120 kJ/m2 UVA. Supplementary Fig. S4: Relative contribution of five mutational signatures reconstructed from point mutations across all conditions of XP‐V complemented (XP‐V_c) and XP‐V cells. (A) Composi [file MC-64-1823-s001.docx]

**Uncovering the role of DNA repair impairment in UVA-induced mutagenesis in human xeroderma pigmentosum variant cells**

Camila Corradi^1^, Natália Cestari Moreno^2^, Nathalia Quintero Ruiz^3^, Giovana da Silva Leandro^1^, Marcela Teatin Latancia^4^, Tiago Antonio de Souza^5^, Veridiana Munford^1^, Carlos Frederico Martins Menck^1*^

**Supplementary material**

**Supplementary Table S1**

Depth of coverage and alignment metrics of XP-V complemented (XP-V_c) and XP-V cells, grouped by treatment and no treatment conditions. NO: untreated; NAC: 10 mM NAC; UVA: 120 kJ/m² UVA; NAC+UVA: 10 mM NAC and 120 kJ/m² UVA.

| **Groups** | **Clones** | **Depth of coverage* (%)** | | | | | | | **Fold enrichment* (x)** | **(%) Aligned reads** | **(%) Target bases** | **(%) Target bp* not covered** | **Mean target coverage*** |
| --- | --- | --- | --- | --- | --- | --- | --- | --- | --- | --- | --- | --- | --- |
|  |  | **1x** | **10x** | **20x** | **30x** | **40x** | **50x** | **100x** |  |  |  |  |  |
| XP-V_c NO | NO-1-c | 99.1 | 97.7 | 92.3 | 83.1 | 72.4 | 61.4 | 19.6 | 60 | 99.9 | 100.0 | 0.0 | 70.96 |
|  | XPVc_50 | 98.8 | 97.4 | 90.4 | 78.1 | 64.3 | 51.6 | 13.6 | 60 | 100.0 | 100.0 | 0.0 | 61.51 |
|  | XPVc_51 | 96.9 | 96.5 | 96.3 | 96.2 | 96.0 | 95.6 | 86.5 | 69 | 100.0 | 99.8 | 0.2 | 192.63 |
|  | XPVc_54 | 96.9 | 96.6 | 96.4 | 96.3 | 96.1 | 95.9 | 89.0 | 69 | 100.0 | 99.8 | 0.2 | 207.10 |
|  | XPVc_66 | 97.0 | 96.6 | 96.4 | 96.2 | 95.9 | 95.2 | 80.8 | 69 | 100.0 | 99.8 | 0.2 | 170.45 |
|  | XPVc_67 | 97.0 | 96.6 | 96.4 | 96.3 | 96.0 | 95.6 | 84.3 | 69 | 100.0 | 99.8 | 0.2 | 184.84 |
| XP-V_c NAC | NAC-2-c | 99.0 | 98.3 | 95.1 | 88.0 | 78.0 | 66.6 | 21.1 | 60 | 99.9 | 100.0 | 0.0 | 74.65 |
|  | NAC-3-c | 99.0 | 96.7 | 86.4 | 69.9 | 52.4 | 36.9 | 4.0 | 60 | 99.9 | 100.0 | 0.0 | 47.16 |
|  | XPVc_52 | 97.0 | 96.6 | 96.4 | 96.3 | 96.1 | 95.6 | 85.9 | 69 | 100.0 | 99.8 | 0.2 | 187.49 |
|  | XPVc_56 | 97.0 | 96.6 | 96.4 | 96.3 | 96.1 | 95.8 | 87.6 | 69 | 100.0 | 99.8 | 0.2 | 199.53 |
|  | XPVc_57 | 97.0 | 96.6 | 96.4 | 96.3 | 96.0 | 95.6 | 85.8 | 69 | 100.0 | 99.8 | 0.2 | 184.30 |
|  | XPVc_70 | 97.0 | 96.7 | 96.5 | 96.3 | 95.9 | 95.1 | 81.5 | 69 | 100.0 | 99.8 | 0.2 | 173.70 |
| XP-V_c UVA | UVA-2-c | 99.0 | 98.3 | 95.3 | 88.2 | 77.9 | 66.3 | 18.4 | 60 | 99.9 | 100.0 | 0.0 | 71.61 |
|  | XPVc_58 | 97.0 | 96.5 | 96.3 | 96.2 | 96.0 | 95.7 | 88.5 | 69 | 100.0 | 99.8 | 0.2 | 200.58 |
|  | XPVc_60 | 99.0 | 98.5 | 96.7 | 92.1 | 85.0 | 76.4 | 37.7 | 59 | 100.0 | 100.0 | 0.0 | 97.62 |
|  | XPVc_61 | 96.8 | 96.5 | 96.3 | 96.2 | 96.0 | 95.6 | 86.9 | 69 | 100.0 | 99.8 | 0.2 | 194.31 |
|  | XPVc_72 | 97.0 | 96.6 | 96.4 | 96.3 | 96.1 | 95.8 | 87.3 | 69 | 100.0 | 99.8 | 0.2 | 193.51 |
|  | XPVc_73 | 96.9 | 96.6 | 96.4 | 96.3 | 96.1 | 95.8 | 89.7 | 69 | 100.0 | 99.8 | 0.2 | 219.45 |
| XP-V_c NAC+UVA | UNAC-1-c | 98.9 | 98.3 | 95.6 | 88.6 | 77.8 | 65.1 | 15.9 | 60 | 99.9 | 100.0 | 0.0 | 68.91 |
|  | UNAC-3-c | 99.0 | 98.3 | 95.1 | 88.1 | 78.4 | 67.6 | 22.2 | 60 | 99.9 | 100.0 | 0.0 | 75.30 |
|  | XPVc_62 | 96.9 | 96.6 | 96.4 | 96.3 | 96.1 | 95.7 | 87.3 | 69 | 100.0 | 99.8 | 0.2 | 194.22 |
|  | XPVc_63 | 96.9 | 96.6 | 96.4 | 96.3 | 96.1 | 95.7 | 85.7 | 69 | 100.0 | 99.8 | 0.2 | 185.70 |
|  | XPVc_64 | 96.9 | 96.6 | 96.4 | 96.2 | 96.0 | 95.5 | 85.6 | 69 | 100.0 | 99.8 | 0.2 | 186.24 |
|  | XPVc_75 | 99.0 | 98.5 | 97.0 | 92.8 | 85.9 | 77.4 | 37.7 | 60 | 100.0 | 100.0 | 0.0 | 97.65 |
| XP-V NO | NO_1 | 98.6 | 95.8 | 89.1 | 80.7 | 72.1 | 63.7 | 29.8 | 61 | 100.0 | 100.0 | 0.0 | 80.29 |
|  | NO_2 | 98.9 | 97.6 | 91.2 | 79.8 | 66.4 | 53.2 | 11.2 | 60 | 100.0 | 100.0 | 0.0 | 59.86 |
|  | NO_4 | 99.0 | 96.8 | 88.5 | 76.8 | 64.8 | 53.7 | 16.2 | 60 | 100.0 | 100.0 | 0.0 | 64.22 |
|  | XPV_36 | 98.9 | 98.0 | 94.4 | 86.8 | 77.0 | 66.7 | 28.1 | 59 | 100.0 | 100.0 | 0.0 | 81.97 |
|  | XPV_37 | 98.9 | 98.1 | 94.3 | 86.6 | 76.8 | 66.6 | 29.2 | 59 | 100.0 | 100.0 | 0.0 | 84.12 |
|  | XPV_38 | 98.9 | 98.2 | 95.6 | 89.3 | 80.5 | 70.8 | 32.5 | 59 | 100.0 | 100.0 | 0.0 | 89.47 |
| XP-V NAC | NAC_1 | 98.9 | 98.0 | 93.7 | 85.0 | 73.8 | 62.0 | 17.8 | 60 | 100.0 | 100.0 | 0.0 | 68.95 |
|  | NAC_2 | 98.9 | 98.3 | 95.4 | 88.8 | 79.4 | 68.6 | 21.9 | 60 | 100.0 | 100.0 | 0.0 | 74.97 |
|  | NAC_3 | 98.9 | 98.2 | 94.8 | 87.4 | 77.3 | 66.1 | 19.9 | 60 | 100.0 | 100.0 | 0.0 | 72.20 |
|  | XPV_39 | 98.8 | 98.1 | 95.4 | 89.4 | 80.9 | 71.4 | 32.3 | 59 | 100.0 | 100.0 | 0.0 | 88.93 |
|  | XPV_40 | 98.9 | 98.2 | 95.4 | 89.0 | 80.0 | 70.3 | 31.5 | 59 | 100.0 | 100.0 | 0.0 | 87.93 |
|  | XPV_41 | 98.9 | 98.1 | 94.3 | 86.8 | 77.2 | 67.2 | 29.6 | 60 | 100.0 | 100.0 | 0.0 | 84.71 |
| XP-V UVA | UVA1 | 98.9 | 97.8 | 93.2 | 84.7 | 74.3 | 63.4 | 21.1 | 59 | 100.0 | 100.0 | 0.0 | 72.72 |
|  | UVA3 | 98.9 | 97.4 | 91.8 | 82.3 | 71.1 | 59.9 | 18.9 | 59 | 100.0 | 100.0 | 0.0 | 69.14 |
|  | UVA4 | 98.9 | 98.1 | 94.7 | 86.9 | 76.0 | 63.6 | 14.7 | 60 | 100.0 | 100.0 | 0.0 | 66.96 |
|  | XPV_44 | 98.9 | 98.2 | 94.8 | 87.5 | 77.9 | 67.9 | 30.1 | 59 | 100.0 | 100.0 | 0.0 | 85.62 |
|  | XPV_45 | 98.9 | 98.3 | 96.1 | 90.9 | 83.1 | 74.2 | 36.0 | 59 | 100.0 | 100.0 | 0.0 | 94.82 |
|  | XPV_46 | 98.8 | 98.0 | 94.6 | 87.3 | 77.4 | 67.0 | 26.9 | 59 | 100.0 | 100.0 | 0.0 | 80.95 |
| XP-V NAC+UVA | UNAC_1 | 98.9 | 97.9 | 92.8 | 82.9 | 70.6 | 57.7 | 13.5 | 60 | 100.0 | 100.0 | 0.0 | 63.67 |
|  | UNAC_2 | 98.9 | 98.3 | 96.1 | 90.6 | 81.9 | 71.2 | 22.1 | 60 | 100.0 | 100.0 | 0.0 | 75.77 |
|  | UNAC_3 | 98.9 | 98.1 | 94.4 | 86.3 | 75.5 | 63.7 | 17.6 | 60 | 100.0 | 100.0 | 0.0 | 69.29 |
|  | XPV_47 | 98.9 | 98.2 | 95.1 | 88.3 | 79.0 | 69.1 | 30.7 | 59 | 100.0 | 100.0 | 0.0 | 86.45 |
|  | XPV_48 | 98.9 | 98.3 | 95.7 | 90.1 | 82.0 | 73.1 | 35.7 | 59 | 100.0 | 100.0 | 0.0 | 94.86 |
|  | XPV_49 | 98.9 | 98.3 | 95.8 | 89.9 | 81.5 | 72.1 | 33.7 | 59 | 100.0 | 100.0 | 0.0 | 90.77 |
|  | XPVUVANAC4 | 97.0 | 96.7 | 96.4 | 95.9 | 94.6 | 92.3 | 67.7 | 62 | 100.0 | 99.8 | 0.2 | 151.05 |
|  | XPVUVANAC5 | 97.0 | 96.7 | 96.4 | 96.0 | 94.8 | 92.8 | 68.7 | 62 | 100.0 | 99.8 | 0.2 | 150.82 |
|  | XPVUVANAC6 | 97.0 | 96.6 | 96.4 | 95.8 | 94.4 | 91.9 | 65.1 | 62 | 100.0 | 99.8 | 0.2 | 140.74 |
|  | XPVUVANAC735 | 97.0 | 96.6 | 96.4 | 96.0 | 95.1 | 93.3 | 70.1 | 62 | 100.0 | 99.8 | 0.2 | 150.90 |
| * Depth of coverage: number of times the nucleotide was read during sequencing. Fold enrichment: number of times the "bait" region was amplified relative to the genome. bp: base pairs. Mean target coverage: total target base pairs divided by the total size of the target region. | | | | | | | | | | | | | |

**Supplementary Table S2**

Absolute count of mutations on each strand, per group of clones and per mutations type. Base substitutions on the coding (sense) strand were classified as Untranscribed, while those on the opposite strand are Transcribed. Substitutions overlapping gene bodies on both strands were excluded from strand-specific analysis. NO: untreated; NAC: 10 mM NAC; UVA: 120 kJ/m² UVA; NAC+UVA: 10 mM NAC and 120 kJ/m² UVA.

| **Groups** | **Type** | **Transcribed** | **Untranscribed** | **Total** | **P-value** | **Sig** |
| --- | --- | --- | --- | --- | --- | --- |
| XP-V_c NO | C>A | 14 | 13 | 27 | 1.0000 |  |
| XP-V_c NO | C>G | 12 | 4 | 16 | 0.0768 |  |
| XP-V_c NO | C>T | 25 | 24 | 49 | 1.0000 |  |
| XP-V_c NO | T>A | 7 | 6 | 13 | 1.0000 |  |
| XP-V_c NO | T>C | 13 | 15 | 28 | 0.8506 |  |
| XP-V_c NO | T>G | 11 | 3 | 14 | 0.0574 |  |
| XP-V_c NAC | C>A | 36 | 27 | 63 | 0.3135 |  |
| XP-V_c NAC | C>G | 14 | 8 | 22 | 0.2863 |  |
| XP-V_c NAC | C>T | 66 | 56 | 122 | 0.4153 |  |
| XP-V_c NAC | T>A | 11 | 8 | 19 | 0.6476 |  |
| XP-V_c NAC | T>C | 27 | 11 | 38 | 0.0139 | * |
| XP-V_c NAC | T>G | 9 | 3 | 12 | 0.1460 |  |
| XP-V_c UVA | C>A | 25 | 23 | 48 | 0.8854 |  |
| XP-V_c UVA | C>G | 11 | 16 | 27 | 0.4421 |  |
| XP-V_c UVA | C>T | 69 | 97 | 166 | 0.0358 | * |
| XP-V_c UVA | T>A | 12 | 6 | 18 | 0.2379 |  |
| XP-V_c UVA | T>C | 20 | 20 | 40 | 1.0000 |  |
| XP-V_c UVA | T>G | 4 | 8 | 12 | 0.3877 |  |
| XP-V_c NAC+UVA | C>A | 25 | 20 | 45 | 0.5515 |  |
| XP-V_c NAC+UVA | C>G | 16 | 10 | 26 | 0.3269 |  |
| XP-V_c NAC+UVA | C>T | 77 | 60 | 137 | 0.1714 |  |
| XP-V_c NAC+UVA | T>A | 10 | 6 | 16 | 0.4545 |  |
| XP-V_c NAC+UVA | T>C | 17 | 13 | 30 | 0.5847 |  |
| XP-V_c NAC+UVA | T>G | 10 | 11 | 21 | 1.0000 |  |
| XP-V NO | C>A | 37 | 28 | 65 | 0.3211 |  |
| XP-V NO | C>G | 7 | 9 | 16 | 0.8036 |  |
| XP-V NO | C>T | 33 | 43 | 76 | 0.3019 |  |
| XP-V NO | T>A | 6 | 3 | 9 | 0.5078 |  |
| XP-V NO | T>C | 18 | 10 | 28 | 0.1849 |  |
| XP-V NO | T>G | 7 | 5 | 12 | 0.7744 |  |
| XP-V NAC | C>A | 25 | 33 | 58 | 0.3581 |  |
| XP-V NAC | C>G | 7 | 11 | 18 | 0.4807 |  |
| XP-V NAC | C>T | 44 | 35 | 79 | 0.3682 |  |
| XP-V NAC | T>A | 7 | 5 | 12 | 0.7744 |  |
| XP-V NAC | T>C | 16 | 7 | 23 | 0.0931 |  |
| XP-V NAC | T>G | 4 | 3 | 7 | 1.0000 |  |
| XP-V UVA | C>A | 94 | 131 | 225 | 0.0162 | * |
| XP-V UVA | C>G | 26 | 30 | 56 | 0.6889 |  |
| XP-V UVA | C>T | 525 | 871 | 1396 | 1.68×10^-20^ | *** |
| XP-V UVA | T>A | 39 | 65 | 104 | 0.0138 | * |
| XP-V UVA | T>C | 37 | 55 | 92 | 0.0758 |  |
| XP-V UVA | T>G | 18 | 21 | 39 | 0.7493 |  |
| XP-V NAC+UVA | C>A | 126 | 118 | 244 | 0.6541 |  |
| XP-V NAC+UVA | C>G | 24 | 32 | 56 | 0.3497 |  |
| XP-V NAC+UVA | C>T | 460 | 733 | 1193 | 2.59×10^-15^ | *** |
| XP-V NAC+UVA | T>A | 58 | 51 | 109 | 0.5657 |  |
| XP-V NAC+UVA | T>C | 51 | 75 | 126 | 0.0400 | * |
| XP-V NAC+UVA | T>G | 26 | 20 | 46 | 0.4614 |  |


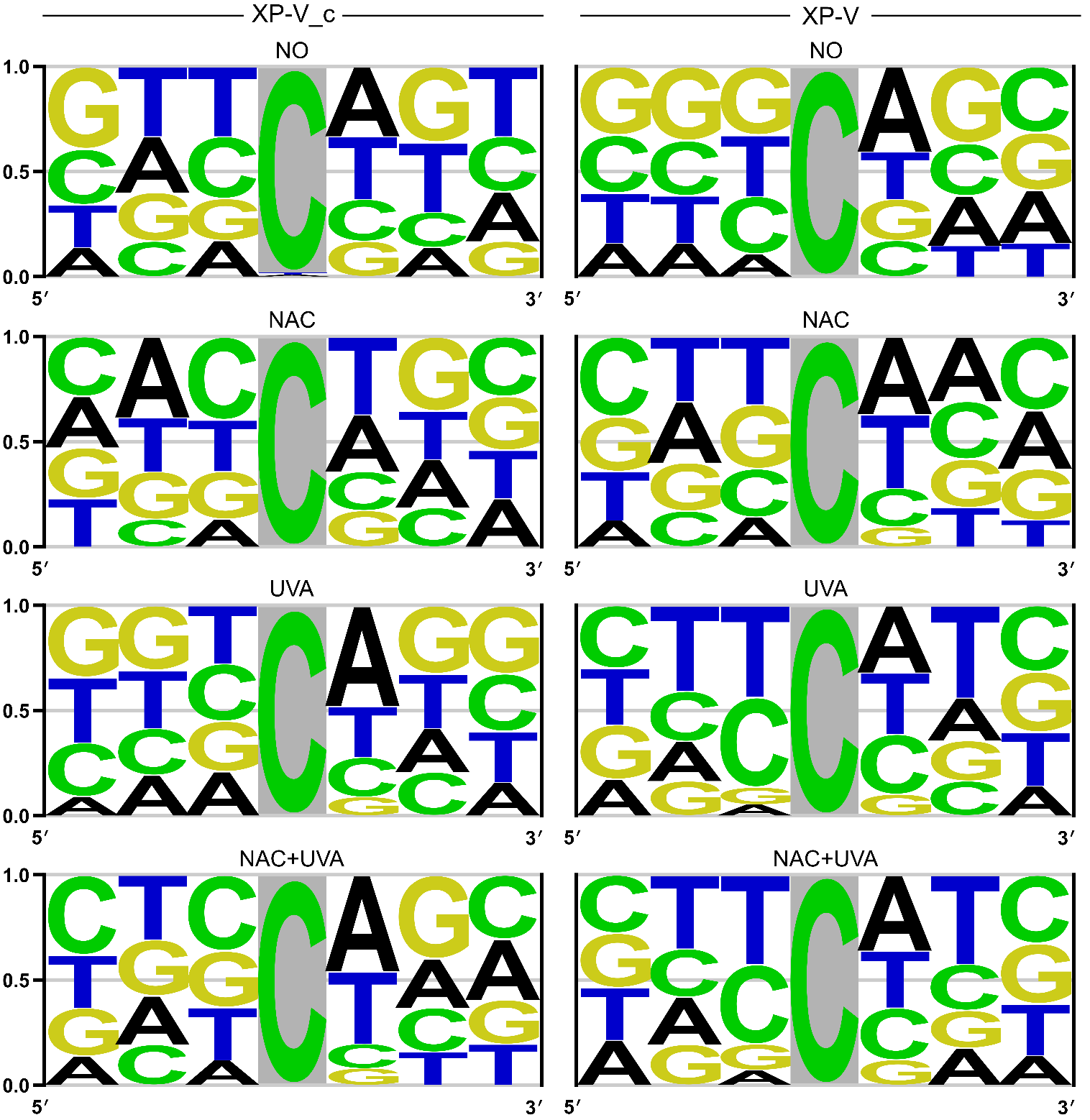


**Supplementary Fig. S1. Frequency of context sequences surrounding C>A mutations detected in all conditions of XP-V complemented (XP-V_c) and XP-V cells.** The enoLOGOS tool was used to examine the frequency of context sequences adjacent to C>A mutations, which are highlighted with a gray background. The human exome sequence served as the background, and the aligned sequences were used to construct the logo. NO: untreated; NAC: 10 mM NAC; UVA: 120 kJ/m² UVA; NAC+UVA: 10 mM NAC and 120 kJ/m² UVA.

**
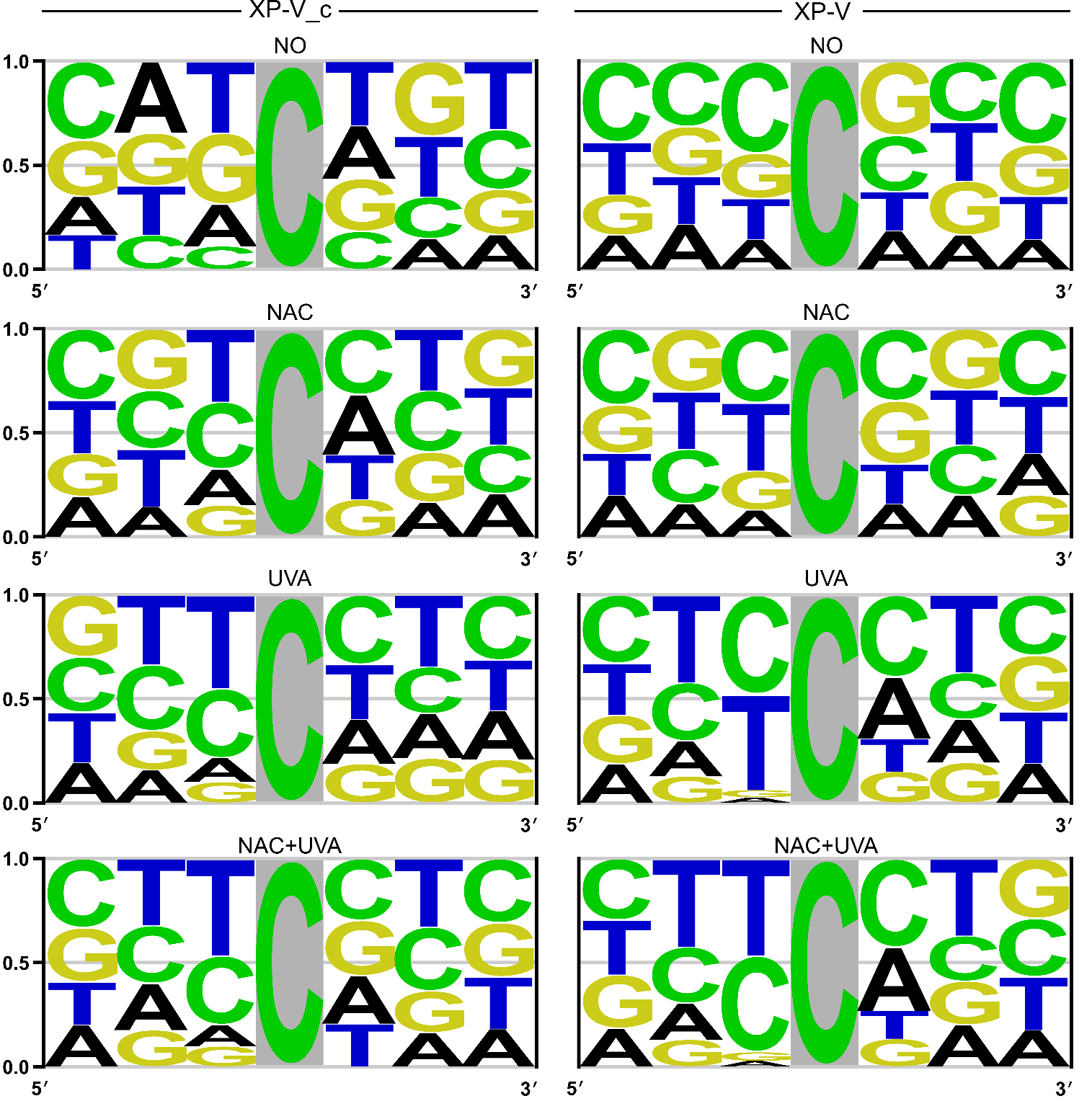
**

**Supplementary Fig. S2. Frequency of context sequences surrounding C>T mutations detected in all conditions of XP-V complemented (XP-V_c) and XP-V cells.** The enoLOGOS tool was used to examine the frequency of context sequences adjacent to C>T mutations, which are highlighted with a gray background. The human exome sequence served as the background, and the aligned sequences were used to construct the logo. NO: untreated; NAC: 10 mM NAC; UVA: 120 kJ/m² UVA; NAC+UVA: 10 mM NAC and 120 kJ/m² UVA.

**
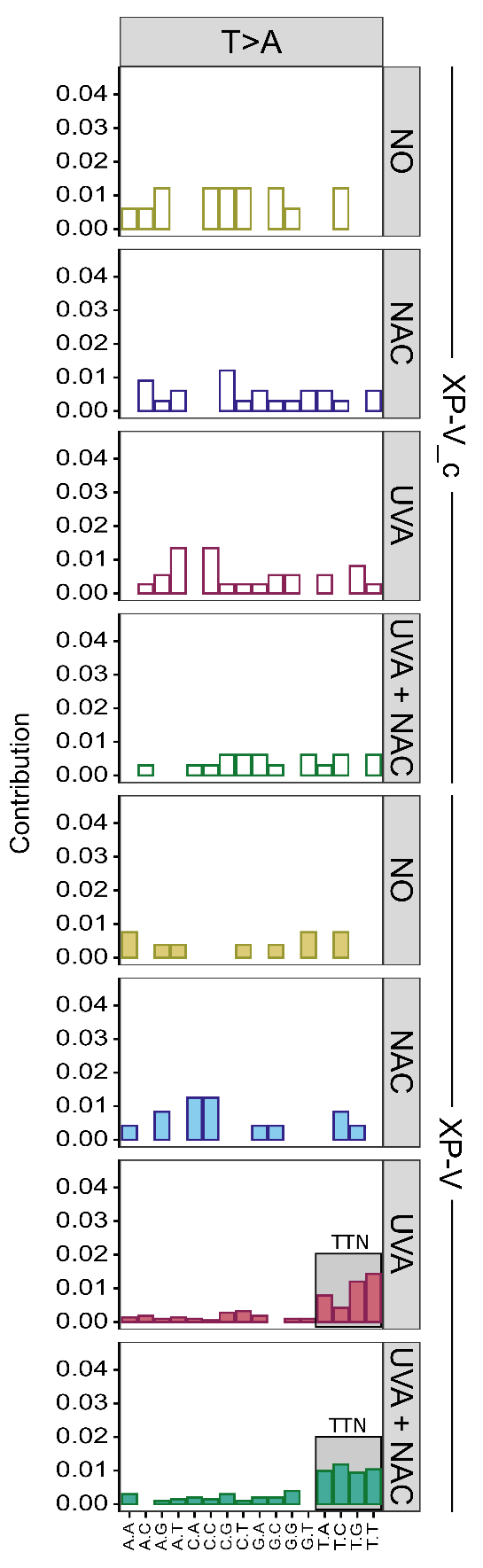
Supplementary Fig. S3. T>A point mutations somatic spectra of XP-V complemented and XP-V cells in detail.** The contribution of somatic spectra only for T>A point mutations distributed in a trinucleotide context for complemented strain (XP-V_c) (shown in lighter colors, first four rows) and XP-V strain (shown in darker colors, last four rows), untreated (NO), treated with NAC, irradiated with UVA, and treated with NAC prior to UVA exposure (NAC+UVA). The contribution represents the mutation frequency for each type of point mutation for each clone set, categorized by lineage and treatment type. On the lower x-axis, the dot should be replaced by T>A mutation. According to IUPAC, N is replaced by A, C, G, or T. NO: untreated; NAC: 10 mM NAC; UVA: 120 kJ/m² UVA; NAC+UVA: 10 mM NAC and 120 kJ/m² UVA.

**
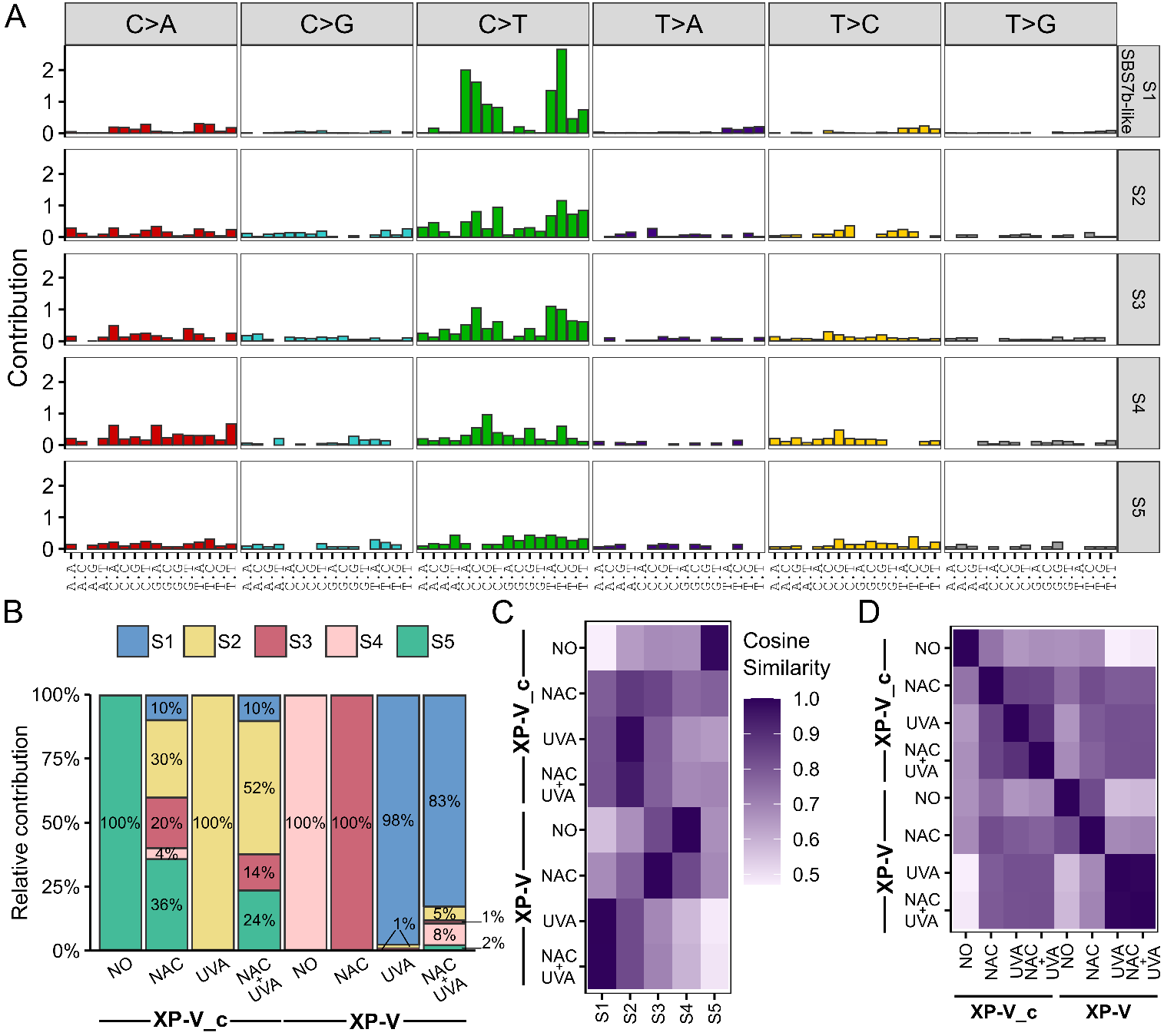
**

**Supplementary Fig. S4. Relative contribution of five mutational signatures reconstructed from point mutations across all conditions of XP-V complemented (XP-V_c) and XP-V cells.** (A) Composition of the mutational spectra for signatures S1, S2, S3, S4, and S5, reconstructed by non-negative matrix factorization (NMF). Signature S1 shows a high cosine similarity (> 0.88) with the SBS7b signature (UV exposure) from COSMIC and is therefore identified as SBS7b-like. (B) Graphical representation of the contribution of each reconstructed signature to the mutational spectrum of the clones. (C) Cosine similarity between the estimated signatures and the mutational profile of each clone set. (D) Cosine similarity between the mutational profiles of each clone set. NO: untreated; NAC: 10 mM NAC; UVA: 120 kJ/m² UVA; NAC+UVA: 10 mM NAC and 120 kJ/m² UVA.
